# Life with Boron: Steroid Architecture and the Chemistry of Marine Boronosteroids

**DOI:** 10.3390/md24030113

**Published:** 2026-03-19

**Authors:** Valery M. Dembitsky, Alexander O. Terent’ev, Sergey V. Baranin, Romulus I. Scorei

**Affiliations:** 1 Bio-Pharm Laboratories, 23615 El Toro Rd X, Lake Forest, CA 92630, USA; 2 Zelinsky Institute of Organic Chemistry, Russian Academy of Sciences, 47 Leninsky Prospect, Moscow 119334, Russia; terentev@ioc.ac.ru (A.O.T.); svbar@ioc.ac.ru (S.V.B.); 3 Department of Biochemistry, BioBoron Research Institute, Natural Research, 31B Dunării Street, 207465 Podari, Romania; romulus_ion@yahoo.com

**Keywords:** marine, invertebrates, diol, boron, borates, steroids, boronosteroids, activity

## Abstract

Marine invertebrates produce a remarkable diversity of polyhydroxylated steroids and secosteroids whose structural features—particularly vicinal (1,2-)diols, 1,3-diols, and clustered hydroxyl arrays—make them well suited for coordination with boron species. In the marine environment, where boron is abundant, chemically stable, and predominantly present as borate under mildly alkaline conditions, such interactions are not only plausible but may be widespread. This review examines the chemistry of boron–steroid complexation in marine systems, emphasizing how rigid steroidal frameworks preorganize diol motifs to form reversible yet stable borate esters under environmentally relevant conditions. We discuss how polyhydroxy steroids may exist in dynamic equilibria between free and boron-bound forms, with speciation governed by pH, boron concentration, and local microenvironmental factors rather than enzymatic control. Boron complexation can modulate key physicochemical properties, including solubility, conformation, and membrane affinity, thereby influencing the biological activity of marine steroids without covalent modification of the carbon framework. By integrating examples from sponges, echinoderms, and corals together with well-characterized model polyols, this review highlights boron complexation as an underrecognized but potentially important factor influencing the structure, function, and bioactivity of marine steroid metabolites.

## 1. Introduction

Marine invertebrates are a rich source of structurally diverse steroids and secosteroids, many of which are distinguished by an unusually high density of hydroxyl groups. Polyhydroxy steroids isolated from sponges, echinoderms, corals, fungal endophytes, and related taxa frequently contain vicinal 1,2-diols, clustered hydroxyl arrays, or 1,3-diols embedded in rigid steroidal frameworks [1,2,3,4]. These structural motifs are well known in chemistry to engage in strong, reversible complexation with boron species, particularly borates and, under certain conditions, boric acid. Despite this, the potential role of boron–steroid complexation in marine systems has received little systematic attention.

Seawater represents a chemically distinctive environment in which such interactions are especially plausible. Boron is present in seawater at relatively high and stable concentrations (hundreds of micromolar), orders of magnitude higher than in most terrestrial biological fluids. At the slightly alkaline pH typical of marine environments, a significant fraction of dissolved boron exists as tetrahedral borate, the form most competent for binding cis-diols [3,4,5,6,7,8]. In addition, marine sediments and geological deposits enriched in boron minerals can locally elevate boron availability far beyond open-ocean background levels. Within these settings, polyhydroxy steroids produced by marine invertebrates are continuously exposed to boron species under conditions that favor complex formation [9,10,11,12,13,14].

From a chemical perspective, the interaction between boron and polyhydroxy steroids is both straightforward and versatile. Vicinal diols readily form cyclic borate esters, while closely spaced hydroxyl groups on rigid scaffolds can generate chelated boron complexes with enhanced stability due to preorganization and reduced entropic penalties. Steroids bearing multiple diol motifs may therefore exist not as a single molecular entity, but as dynamic ensembles of free and boron-bound species whose distribution depends on local pH, boron concentration, and competing ligands. In contrast to enzymatically generated conjugates such as sulfates or glycosides, boron complexation is non-enzymatic, reversible, and highly sensitive to environmental conditions, making it particularly well suited to marine habitats [15,16,17,18,19,20].

The biological implications of this chemistry are potentially significant. Many marine polyhydroxy steroids exhibit antiviral, cytotoxic, anti-inflammatory, or signaling activities, yet their potency and selectivity often vary markedly with extraction conditions, assay media, or purification protocols [1,2,3,4,21,22,23,24]. Boron complexation offers a plausible, and largely unexplored, explanation for some of this variability. Complex formation can alter solubility, membrane affinity, conformational preferences, and effective concentration of the free steroid, thereby modulating bioactivity without altering the covalent structure of the molecule. In boron-rich microenvironments—such as sponge tissues, mucus layers, or sediment-associated niches—boron–steroid complexes may serve as storage forms, transport intermediates, or chemically buffered reservoirs of bioactive metabolites [25,26,27,28,29,30].

In this review, we explore the concept that polyhydroxy steroids from marine invertebrates are predisposed to form stable boron complexes under environmentally relevant conditions. Using well-characterized model systems, such as ouabain and other polyol-rich steroids, alongside examples from sponges and echinoderms, we examine the structural requirements for boron binding, the types of complexes that can form, and the factors governing their stability and speciation. By integrating insights from marine chemistry, natural products chemistry, and coordination chemistry, this review aims to highlight boron complexation as an underappreciated but potentially important dimension of marine steroid biology and bioactivity.

## 2. Ouabain Boron Complexes as a Model for Studying Polyhydroxy Steroids

Ouabain (g-strophanthin) is a naturally occurring cardiac glycoside isolated mainly from African plants of the genera *Strophanthus* and *Acokanthera*. It was historically used as an arrow poison because of its strong cardiotoxic activity [31,32,33,34]. At the molecular level, ouabain is a specific inhibitor of Na^+^/K^+^-ATPase, the membrane enzyme responsible for maintaining sodium and potassium gradients across the plasma membrane. Inhibition of this pump increases intracellular sodium, which secondarily elevates intracellular calcium via the Na^+^/Ca^2+^ exchanger and enhances cardiac contractility [33,34,35,36,37]. Beyond its classical cardiotonic action, ouabain has also become important in physiology and pharmacology as a tool for studying ion transport, signal transduction, inflammation, apoptosis, and cancer-related signaling [38,39,40,41,42,43].

### 2.1. Ouabain–Boron Complexes: Rationale, Synthesis, and Potential Biological Activity

Ouabain contains numerous hydroxyl groups in both its steroidal aglycone and sugar moiety, creating several polyol and diol sites that may coordinate boron. This makes ouabain a plausible ligand for boric acid or borate ions, which are well known to form reversible cyclic esters with vicinal hydroxyl groups. Although such reactions are common for carbohydrates and glycosides, they have been studied much less for cardiac glycosides such as ouabain [44,45,46,47,48]. Formation of ouabain–boron complexes can be explained by the Lewis acidity of boron, which allows it to bind neighboring oxygen atoms and generate cyclic borate esters. In ouabain, these interactions may occur within the sugar residue or at suitable hydroxyl pairs on the steroid nucleus. Such complexation could modify the molecule’s polarity, conformation, and hydrogen-bonding properties, and may therefore influence its interaction with Na^+^/K^+^-ATPase and other biological targets. For this reason, ouabain is a useful model compound for studying boron coordination in polyhydroxy steroids, with possible relevance to medicinal chemistry and structure–activity relationships.

### 2.2. Chemical Basis for Ouabain–Boron Complexation

Boron typically coordinates to oxygen-rich substrates through trigonal boric acid [B(OH)_3_] or tetrahedral borate [B(OH)_4_^−^], forming mono- or bicyclic borate esters. In ouabain, several candidate binding sites exist, including the cis-diol units of the rhamnose sugar and hydroxylated positions on the steroid nucleus. Under mildly alkaline aqueous conditions, borate ions could chelate these diols, generating neutral or anionic ouabain–borate complexes. Such complexes would be dynamic, pH-dependent, and potentially stabilized by intramolecular hydrogen bonding [44,49].

**Figure 1:**
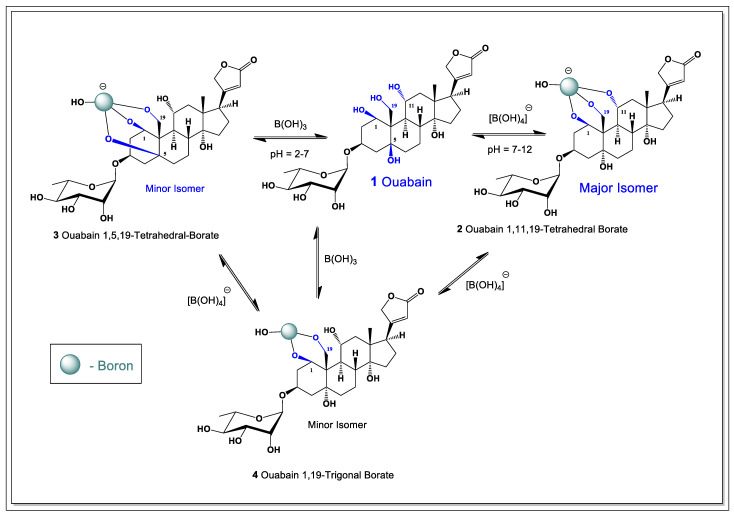
The general scheme for the formation of boron complexes with ouabain is as follows. Borax, when dissolved in water, forms boric acid, and boron anion reacts with quabain and forms tetrahedral- and trigonal-borate complexes. Highlighted in blue are hydroxyl groups that interact with boric acid or boron anion to form cycloborates.

Within the ouabagenin framework, only two tri-hydroxyl combinations can geometrically accommodate a tetrahedral borate: the 1,11,19-hydroxyl and 1,5,19-hydroxyl arrangements. NMR analysis revealed that the major isomer corresponds to the 1,11,19-borate complex, in which borate coordination stabilizes ring A in a boat-like conformation. In contrast, nuclear Overhauser effect (NOE) correlations observed for the minor isomer are consistent with the 1,5,19-borate arrangement, which locks ring A into a chair conformation (Figure 1).

This cyclic borate protection of the 1,11,19- or 1,5,19-hydroxyl groups has important chemical consequences. Specifically, nanogram-scale naphthoylation experiments performed on glassware-treated ouabain failed to produce the expected fully derivatized ouabain 1,5,11,14,19-pentanaphthoate, indicating that borate complexation sterically and electronically shields key hydroxyl groups from acylation.

### 2.3. Plausible Synthetic Approaches

The synthesis of ouabain–boron complexes would not require covalent modification of the steroid scaffold and could proceed under mild conditions. A representative approach would involve incubating ouabain with boric acid or a borate salt (e.g., sodium borate) in aqueous or mixed aqueous–organic solvents (water–methanol or water–DMSO) at controlled pH (typically 8–10). Complex formation could be monitored using ^11^B NMR spectroscopy, FTIR (B–O stretching modes), mass spectrometry, and changes in chromatographic behavior. Importantly, such complexation would be reversible, allowing isolation either as discrete complexes or as equilibrium mixtures depending on solvent and pH [44,47,50].

### 2.4. Potential Biological Consequences

Complexation of ouabain with boron may modify key physicochemical properties, including solubility, charge distribution, and hydrogen-bonding capacity. Such changes could influence its interaction with Na^+^/K^+^-ATPase, potentially affecting binding affinity, isoform selectivity, or signaling pathways. Borate complex formation may also alter membrane interactions, cellular uptake, and tissue distribution, leading to changes in pharmacological activity or toxicity profiles [51,52,53]. In addition, boron is increasingly recognized as a biologically relevant element capable of influencing enzyme activity and cell signaling, suggesting possible modulatory effects when combined with a bioactive molecule such as ouabain.

At present, ouabain–boron complexes remain hypothetical but chemically plausible structures. Experimental studies are needed to verify their formation, stability, and biological properties. Future work combining synthetic chemistry, structural characterization, and biological testing may clarify whether boron complexation can modulate the activity or selectivity of cardiac glycosides. Such studies could contribute to the broader exploration of boron-assisted modulation of natural product bioactivity in medicinal chemistry.

### 2.5. Activity of Ouabain–Boron Complexes

**Figure 2:**
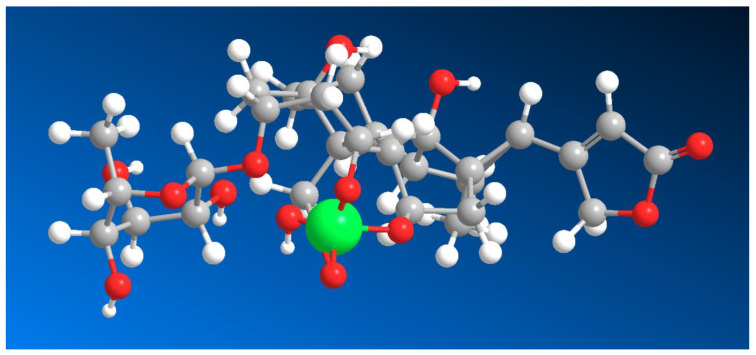
3D graph of the complex of the major isomer ouabain 1,11,19-tetrahedral borate (**2**). The boron atom is highlighted in green, and oxygen is highlighted in red.

**Figure 3:**
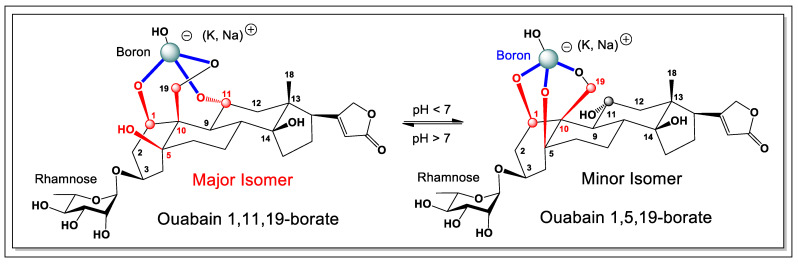
Three-dimensional structures of two coordination isomers of tetrahedral ouabain borates. The chemical nature of the transition from the major to the minor isomer has not been established. It is assumed that this may occur due to the position of the hydroxyl groups, changes in pH, or other factors.

The formation of boron complexes with ouabain (see Figure 2 and Figure 3) significantly influences both its chemical behavior and biological activity. Ouabain exerts its primary pharmacological effects through high-affinity inhibition of the Na^+^/K^+^-ATPase, leading to elevated intracellular Na^+^ levels and secondary increases in Ca^2+^ via the Na^+^/Ca^2+^ exchanger. Coordination of borate to the aglycone hydroxyl groups modifies the conformational landscape of ouabain, particularly affecting ring A geometry, which is known to play a role in enzyme binding [44,50,51,52,53,54].

Tetrahedral borate coordination involving three hydroxyl groups (predominantly at the 1,11,19-positions) rigidifies the steroid core and alters hydrogen-bonding capabilities critical for interaction with Na^+^/K^+^-ATPase. Such structural modulation is expected to affect binding kinetics and possibly reduce immediate inhibitory potency while stabilizing the molecule against chemical degradation. In contrast, trigonal borate complexes, formed under acidic conditions, represent more weakly coordinated species that readily interconvert with free ouabain, preserving biological activity [44,50].

Importantly, the borate–ouabain interaction is reversible and pH-dependent, suggesting a dynamic equilibrium between free and boron-complexed forms in biological environments. This behavior supports the concept that boron complexes may function as transient or reservoir forms of ouabain, modulating its bioavailability, cellular uptake, and duration of action rather than acting as permanently inactivated species.

Additionally, cyclic borate protection of hydroxyl groups reduces susceptibility to metabolic or chemical derivatization, which may influence pharmacokinetic properties such as stability and distribution. Although direct comparative biological assays of isolated ouabain–boron complexes remain limited, available structural and spectroscopic data strongly suggest that borate coordination fine-tunes, rather than abolishes, ouabain’s biological function [44,54,55].

Overall, ouabain–boron complexes exemplify how reversible inorganic coordination can modulate the activity of a potent natural product, highlighting boron chemistry as a subtle regulatory element in cardiac glycoside pharmacology and offering a framework for designing tunable ouabain derivatives with controlled activity profiles.

## 3. Ouabain vs. Marine Polyhydroxysteroids

Below are two complementary angles: (i) how ouabain compares to “sugar-free” marine polyhydroxysteroids, and (ii) a compact equilibrium/speciation model for boron–steroid complexation in seawater (including what knobs control whether the complex is a trace curiosity vs. a dominant form).

### 3.1. Ouabain vs. Marine Polyhydroxysteroids That Lack Sugars

The structural “boron binding capacity” of ouabain has been demonstrated experimentally [44,45,46,47,48]. Ouabain is unusually predisposed to boron complexation because it has two kinds of boron-binding motif: (i) A glycone (sugar) with multiple cis-diols → borate’s favorite substrate (high probability of forming 5-membered cyclic borate esters); (ii) A polyhydroxylated aglycone (steroid core) with several –OH groups → potential additional chelation sites, though typically weaker unless they present a true vicinal/cis-diol.

By contrast, many sugar-free marine polyhydroxysteroids have: (i) Multiple hydroxyls on the steroid nucleus, but fewer true cis-diol pairs than a carbohydrate does, so their intrinsic borate affinity can be lower (unless the hydroxyl pattern happens to be vicinal and correctly oriented).

#### 3.1.1. Representative “Sugar-Free” Marine Steroid Families

Marine natural products literature is packed with polar, oxygenated steroids that are not glycosides, for example: (i) Polyhydroxylated steroids from corals/gorgonians (aglycones; no sugar) [1,2,3,4]; (ii) “Marine polar steroids” including notable non-glycosidic scaffolds like contignasterol and squalamine (aminosterol) [13,14]; (iii) Highly oxygenated/steroid-derived cytotoxins (e.g., cephalostatins/ritterazines class), again not sugar glycosides [56,57].

And in echinoderms specifically, surveys show a mixture of: (i) Asterosaponins/steroid glycosides (with sugars) [58,59]; (ii) Polyhydroxylated steroids (some without sugars) detected alongside them [1,2,3,4,12,24,60,61].

#### 3.1.2. Net Prediction for Boron Complexation in Seawater

If you put these side-by-side in seawater: (i) Ouabain: most likely to form boron complexes at measurable fractions because sugar diols make “good handles” for borate; (ii) Sugar-free polyhydroxysteroids: complexation can still occur, but it will be highly dependent on the exact hydroxyl topology (do you actually have a cis-vicinal diol, or just scattered hydroxyls?). Many will bind boron more weakly than a glycoside unless they contain an especially favorable diol arrangement.

A key ecological nuance: even a “weak binder” can become meaningfully complexed when boron is abundant (seawater) and the steroid is locally concentrated (membranes, glands, vesicles, defensive secretions).

#### 3.1.3. Sketching Equilibrium Models for Boron–Steroid Speciation in Seawater

*(a)*  *Step 0—boron acid/base speciation*

In seawater, dissolved boron occurs predominantly as boric acid, B(OH)_3_, and borate, B(OH)_4_^−^. Under seawater conditions, the apparent (conditional) dissociation constant is often cited around pK*a ≈ 8.6 (temperature- and salinity-dependent), so at typical seawater pH (~8.0–8.2), a substantial fraction of total boron is present as borate. Empirically, within ~pH 7.8–8.1, a commonly cited ballpark ratio is B(OH)_3_:B(OH)_4_^−^ ≈ 4:1.

B(OH)_3_ + H_2_O ⇌ B(OH)4^−^ + H^+^

with K*_a_ (conditional at seawater ionic strength). This distinction matters because tetrahedral borate is generally the most competent form for rapid and reversible cis-diol complexation.

$$K_{1}^{*}=\frac{\left[BL^{-}\right]}{\left[B{\left(OH\right)}_{4}^{-}\right][L]}$$

Then the *fraction complexed* (ignoring other boron sinks for the moment) is:

$$f_{L}=\frac{\left[BL^{-}\right]}{[{L]}_{\mathrm{tot}}}=\frac{K_{1}^{*}[B(OH{)}_{4}^{-}]}{1+K_{1}^{*}[B(OH{)}_{4}^{-}]}$$

Then, ignoring other boron sinks, the extent of ligand complexation is governed by the balance between free ligand and borate availability. When the product of the conditional association constant (K_1_*) and the borate concentration increases, the proportion of ligand present in the boron-bound form correspondingly rises, approaching dominance when this product becomes much greater than unity.

*Seawater insight*. Because the concentration of borate, [B(OH)_4_^−^], can reach the order of 10^−4^ M at pH ~8 (total boron ~4 × 10^−4 ^ M, with a substantial borate fraction), even moderate values of K_1_* are sufficient for boron–steroid complexation to become chemically significant under marine conditions.

*(b)*  *Step 1—simplest binding model (1:1 borate–diol complex)*

Let a steroid (or a diol-bearing substructure) be represented as **L** (ligand). A minimal binding model is:

B(OH)_4_^−^ + L ⇌ BL^−^ + H_2_O

where BL^−^ denotes a cyclic borate ester/“ate” complex (net anionic, written here in a simplified form). Define a conditional association constant:

$$K_{1}^{*}=\frac{\left[BL^{-}\right]}{\left[B{\left(OH\right)}_{4}^{-}\right][L]}$$

Then the *fraction complexed* (ignoring other boron sinks for the moment) is:

$$f_{L}=\frac{\left[BL^{-}\right]}{[{L]}_{\mathrm{tot}}}=\frac{K_{1}^{*}[B(OH{)}_{4}^{-}]}{1+K_{1}^{*}[B(OH{)}_{4}^{-}]}$$

Then, ignoring other boron sinks, the extent of ligand complexation is governed by the balance between free ligand and borate availability. When the product of the conditional association constant ($K_{1}^{*}$) and the borate concentration increases, the proportion of ligand present in the boron-bound form correspondingly rises, approaching dominance when this product becomes much greater than unity.

*Seawater insight*. Because the concentration of borate, [B(OH)_4_^−^], can reach the order of 10^−4^ M at pH ~8 (total boron ~4 × 10^−4 ^ M, with a substantial borate fraction), even moderate values of K_1_* are sufficient for boron–steroid complexation to become chemically significant under marine conditions.

*(c)*  *Step 2—include a boric-acid binding channel (optional)*

Some polyols can also bind directly from boric acid:

B(OH)_3_ + L ⇌ BL^−^ + H_2_O

with a conditional association constant K_0_*.

If both channels are included in a minimal, lumped form, an approximate expression is:

$$f_{L}\approx (K_{1}^{*}\cdot B(OH{)}_{4^{-}}+K_{0}^{*}\cdot B(OH{)}_{3}/(1+K_{1}^{*}\cdot B(OH{)}_{4^{-}}+K_{0}^{*}\cdot B(OH{)}_{3})$$

In practice, at bulk seawater pH, the borate channel often dominates effective diol complexation; however, the boric-acid term can be useful in microenvironments where borate fraction is reduced, or where the ligand is an exceptionally strong binder.

*(d)*  *Step 2b—1:2 complex (one boron, two ligands)*

Some systems form 1:2 complexes, in which one boron center bridges two diol ligands:

B(OH)_4_^−^ + 2L ⇌ BL_2_^−^ + H_2_O

with:

$$K_{2}^{*}=\;{{\mathrm{BL}}_{2}}^{-}/{{\left(B(\mathrm{OH}\right)}_{4}}^{-}\cdot L_{2})$$

This becomes relevant when (i) boron availability is high, (ii) ligands are locally concentrated, and (iii) geometry permits two-point attachment.

In practice, at seawater pH, it is often the borate channel that dominates the effective complexation, but including this term lets you handle cases where borate is lower (microenvironments) or the ligand is exceptionally good.

*(e)*  *Step 3—multidentate and multi-site possibilities (relevant to ouabain)*

Ouabain is not a single-diol ligand: it is a multi-site polyol (sugar plus multiple hydroxyl groups), enabling:

*Site competition (multiple 1:1 complexes):*

Different diol sites (L_1_, L_2_, …) may compete for boron binding, yielding multiple 1:1 complex. This can be modeled with site-specific constants $K_{1,1}^{*}$, $K_{1,2}^{*},$ etc., and the observed distribution becomes a weighted mixture of bound forms.

*Intramolecular Chelation (Effective Molarity):*

If two diol sites on the same molecule are spatially reachable by one boron center, an intramolecular chelate effect can arise (often described via an “effective concentration” or “effective molarity”), increasing apparent affinity relative to mono-diol steroid frameworks.

*(f)*  *Step 4—real seawater caveat: bulk competition is low, but microenvironments matter*

A recurring observation in marine chemistry is that complexation of boric acid/borate with simple diols is often not dominant in bulk seawater due to low natural concentrations of free small-molecule diols. However, our scenario differs because we consider: (i) a specific polyol steroid/glycoside that may be locally concentrated; and (ii) microenvironments (mucus layers, vesicles, membranes, gut-like compartments) where local composition, pH, ionic strength, and partitioning can shift equilibria substantially relative to bulk averages.-

A practical control parameter for assessing whether complex formation can be significant is:

$$K_{1}^{*}\;\times \;[{B(OH)}_{4}{]}^{-}]$$

- If $K_{1}^{*}$ [B(OH)_4_^−^] ≪ 1: mostly free steroid (complex is trace).
- If $K_{1}^{*}$ [B(OH)_4_^−^] ∼ 0.1: complex is noticeable.
- If $K_{1}^{*}$ [B(OH)_4_^−^] ≫ 1: complexed form dominates.

Because seawater can supply [B(OH)_4_^−^] at ~10^−4^ M at pH ~8, moderate K_1_* values may already matter—especially for multi-diol ligands such as ouabain, where multi-site binding and chelation can effectively raise apparent affinity.

## 4. Conformation of Steroids and Its Chemical Significance

Steroids are conformationally rigid yet stereochemically rich molecules. Their tetracyclic framework, combined with controlled flexibility in rings A and D, creates a limited but powerful set of three-dimensional shapes [62,63,64]. The distinction between flat, bent, and distorted conformations explains not only differences in biological activity but also why boron complex formation depends so strongly on steroid stereochemistry. Conformation is the structural language through which steroids express both chemical reactivity and biological function [62,63,64,65,66,67].

### 4.1. General Steroid Framework

Steroids are a broad class of natural and synthetic compounds characterized by a rigid tetracyclic carbon skeleton, commonly referred to as the gonane nucleus. This framework consists of: (i) Three fused six-membered rings (rings A, B, and C); (ii) One fused five-membered ring (ring D) [63,64,65,66,67].

This polycyclic system imposes a high degree of conformational restriction, making steroids fundamentally different from acyclic or monocyclic organic molecules. As a result, small stereochemical changes can produce large effects on molecular shape, reactivity, and biological function [62,63,64,65].

The conformation of steroids is therefore central to: (i) Biological activity (e.g., receptor binding); (ii) Physicochemical properties (solubility, polarity); (iii) Coordination behavior, including boron complex formation [64,65,66,67,68].

The steroid nucleus is exceptionally rigid due to: (i) Multiple fused rings; (ii) Predominantly trans-fused ring junctions; (iii) Limited freedom for bond rotation.

In particular: (i) Rings B and C are almost always trans-fused; (ii) This fusion locks both rings into stable chair conformations; (iii) Ring inversion (“ring flip”), common in cyclohexane, is effectively prevented [68,69,70].

### 4.2. Rigidity of the Steroid Skeleton

The steroid nucleus is exceptionally rigid due to: (i) Multiple fused rings; (ii) Predominantly trans-fused ring junctions [62,63,64].

Limited freedom for bond rotation, In particular: (i) Rings B and C are almost always trans-fused; (ii) This fusion locks both rings into stable chair conformations; (iii) Ring inversion (“ring flip”), common in cyclohexane, is effectively prevented.

This rigidity creates a fixed three-dimensional scaffold onto which functional groups are attached with precise spatial orientation. This is a key reason why steroid stereochemistry is so tightly linked to function [63,64,65,66,67,68,69,70].

### 4.3. Conformation of Individual Rings

Conformation of Individual Rings [68,69,70]: (i) Rings A, B, and C (Six-Membered Rings). Most steroid rings behave similarly to substituted cyclohexanes: (i) Chair conformation is the most stable and predominant; (ii) Axial and equatorial substituents are well defined; (iii) Steric strain is minimized in the chair form.

However, ring A shows greater conformational variability than rings B and C.

Ring A Flexibility [71,72]: (i) When fully saturated, ring A typically adopts a chair conformation; (ii) When unsaturated (e.g., Δ^4^-3-keto steroids such as testosterone), ring A often shifts to a half-chair or distorted chair.

This distortion changes [23,68]: (i) The orientation of oxygen-containing functional groups; (ii) The accessibility of neighboring atoms for coordination; (ii) This flexibility is crucial for interactions with enzymes, receptors, and metal centers.

Ring D (Five-Membered Ring) [23,68]: (i) Ring D is the most flexible part of the steroid skeleton; (ii) Five-membered rings cannot adopt a true chair conformation.

Instead, ring D typically adopts: (i) Envelope conformations; (i) Twist conformations.

Because of this flexibility: (i) Substituents at C17 can occupy different spatial positions: (i) Hydroxyl or carbonyl groups at C17 may be oriented toward or away from the steroid plane; (ii) This variability strongly affects coordination geometry in metal or boron complexes.

### 4.4. Ring Junction Stereochemistry

Ring stereochemistry of 5α vs. 5β steroids. One of the most important conformational distinctions in steroids arises from the junction between rings A and B: (i) 5α-Steroids (Trans A/B Fusion); (ii) Rings A and B are trans-fused [73,74].

### 4.5. Three Conformational Classes of Steroids

Based on stereochemistry and ring fusion, steroid molecules can be broadly grouped into three conformational types: (i) Flat (5α-trans-fused steroids); (ii) Extended geometry; (iii) Functional groups often lie on opposite faces; (iv) Favor linear or bidentate coordination modes; (v) Bent (5β-cis-fused steroids).

Curved molecular shape: (i) Functional groups may be brought into closer proximity; (ii) Enables chelation and intramolecular complex formation; (iii) Enhanced ability to adjust geometry during complexation.

### 4.6. Conformation and Boron Complex Formation

The formation of boron complexes depends largely on steroid conformation, for several reasons [44,45,46,47,48]. Boron typically forms: (i) Trigonal planar or tetrahedral complexes; (ii) Strong bonds with oxygen donors (–OH, =O).

Chelation requires: (i) Proper distance; (ii) Correct orientation; (iii) Favorable stereochemistry.

Influence of Steroid Conformation: (i) Rigid backbones limit which atoms can simultaneously coordinate boron; (ii) Flat steroids may present donor atoms too far apart; (iii) Bent steroids can bring hydroxyl or carbonyl groups into chelating proximity.

Ring D flexibility allows adjustment around the boron center, Thus [23,68,73,74]: (i) 5β-steroids often form more stable boron chelates; (ii) Unsaturated or distorted ring systems enhance adaptive binding; (iii) Subtle stereochemical differences dictate whether mono- or bidentate complexes form.

### 4.7. Boron–Diol Coordination Mechanisms

Boron–diol complexation typically involves the formation of cyclic borate esters through condensation between boronic acids and vicinal or suitably oriented hydroxyl groups [44,45,46,47,48,49]. Reaction Scheme 1 (conceptual):

Steroid–(OH)_2_ + R–B(OH)_2_ ⇌ Steroid–O–B–O (five- or six-membered borate ring) + H_2_O

In rigid, flat steroids, diol groups may be poorly aligned, reducing chelation efficiency. In contrast, bent steroid conformations frequently permit favorable O–B–O angles and distances, stabilizing cyclic borate esters. Ring D flexibility further allows fine adjustment of hydroxyl orientations, enhancing complex stability.

### 4.8. Boron–Keto Coordination Mechanisms

Boron–keto interactions commonly involve coordination of boron to carbonyl oxygen atoms, either directly or via enolized forms. In Δ^4^-3-keto steroids, ring A distortion increases carbonyl accessibility and enhances orbital overlap with boron’s empty p-orbital [44,75,76,77,78].

Reaction Scheme 2 (conceptual):

Steroid–C=O + BX_3_ → Steroid–C=O→B complex or

Steroid–enolate + boron reagent → chelated boron–enolate complex

Bent steroid conformations may further enable cooperative interactions between carbonyl and hydroxyl donors, producing mixed O,O-chelates that are inaccessible in more planar analogs.

### 4.9. Implications for Stability and Reactivity

The stability of boron–steroid complexes is dictated not only by electronic factors but also by conformational preorganization. Steroids that present donor atoms in geometries closely matching boron’s preferred coordination environment form stronger, more persistent complexes. These principles explain observed differences in reactivity, selectivity, and binding strength across steroid classes [79,80,81].

### 4.10. Some Conclusions on Boron and Steroid Interaction

Steroid conformation plays a decisive role in boron complex formation. The rigid gonane framework, modulated by ring junction stereochemistry and localized flexibility in rings A and D, produces a limited set of conformations with profound chemical consequences. Boron–diol and boron–keto coordination mechanisms are particularly sensitive to these structural parameters, with bent and distorted steroids generally exhibiting enhanced chelation capabilities. A detailed understanding of steroid conformation is therefore essential for the rational design and interpretation of boron–steroid complexes.

## 5. Polyhydroxy Steroids of Marine Invertebrates and Their Boron Complexes

Marine invertebrates are ideal organisms for studying boron chemistry. They exist at the intersection of high boron availability, intense production of secondary metabolites, and polyol-rich steroid structures [82,83,84,85].

Marine invertebrates are animals without an internal bony skeleton (backbone) that live in salt water. They comprise approximately 98% of all ocean species and exhibit an incredible diversity of forms, from microscopic plankton to giant squid: (i) Sponges (Porifera)—the simplest multicellular organisms that are sedentary and filter feeders. (ii) Echinoderms (Echinodermata)—These have five-ray symmetry and a unique water vascular system. They include starfish, sea urchins, sea cucumbers, and crinoids. (iii) Cnidarians (Cnidaria)—Animals with radial symmetry and stinging cells. These include jellyfish, corals, and sea anemones. (iv) Marine fungal endophytes.

### 5.1. Why Marine Sponges Are a Uniquely Favorable System for Boron–Diol Chemistry

Marine sponges (phylum Porifera) are ancient, multicellular, sessile aquatic animals found globally, primarily in marine environments from shallow to deep waters. They are filter feeders, using specialized flagellated cells (choanocytes) to pump water, oxygen, and nutrients through a porous body structure. They are ecological cornerstones, providing habitats and assisting in nutrient cycling [86,87,88,89].

Marine sponges also host dense and diverse communities of symbiotic microorganisms, including bacteria, archaea, and microalgae, which can constitute a large fraction of their biomass [87,88,89]. These symbionts contribute to primary production, nitrogen fixation, and the biosynthesis of many bioactive secondary metabolites attributed to sponges. From an evolutionary perspective, Porifera represent one of the earliest-branching metazoan lineages, offering key insights into the origins of multicellularity and animal cell differentiation. Sponges reproduce both sexually and asexually, with remarkable regenerative capacities that allow them to recover from fragmentation or environmental stress. Owing to their chemical diversity and ecological resilience, marine sponges have become invaluable models in marine ecology, evolutionary biology, and natural product research [89,90,91,92].

Marine sponges live in [86,88,91]: (i) Constant seawater exposure (~0.4–0.5 mM total boron); (ii) pH ~8.0–8.2, which significantly increases the fraction of borate B(OH)_4_^−^; (iii) Confined microenvironments (mesohyl, canals, mucus layers) where ions and metabolites concentrate.

Unlike vertebrate plasma, this is a system where boron–diol equilibria are shifted toward complex formation by default: (i) Sponge steroids and metabolites: 1,2-diols are everywhere; (ii) Sponges are famous for producing highly oxygenated steroids and related metabolites, often with 1,2-diol (vicinal diol) motifs; (iv) Multiple hydroxyls on rigid polycyclic scaffolds (little or no glycosylation unlike ouabain); (v) Secosteroids and rearranged steroids with adjacent hydroxyls; (vi) Oxygenated terpenoids and polyketide–steroid hybrids.

Sea sponges are a rich source of structurally diverse steroids, including compounds bearing 1,3-diol functional motifs. These 1,3-diols are of particular chemical interest because they can readily coordinate with boron atoms to form stable cyclic borate complexes. Such boron complexation can significantly alter the physicochemical properties of the parent steroids, including their stability, lipophilicity, and biological activity. Consequently, sponge-derived 1,3-diol steroids represent promising scaffolds for the development of novel boron-containing bioactive molecules [1,2,3,4,21,22,23,80,81].

#### 5.1.1. Stelletasterenol as a Boron-Coordination-Capable Secosteroid

Stelletasterenol (**5**) is a structurally unusual cyclic 9(11)-secosterenol isolated from the marine sponge *Euryspongia arenaria*. Unlike classical secosteroids formed by simple C–C bond cleavage, stelletasterenol features an oxygen bridge between C-9 and C-11, generating a rare cyclic ether-containing secosteroid architecture. This distinctive structural motif confers a unique three-dimensional topology and has been linked to its biological activity as a platelet-activating factor (PAF) agonist, implicating stelletasterenol in inflammatory and thrombotic signaling pathways [14,93].

**Figure 4:**
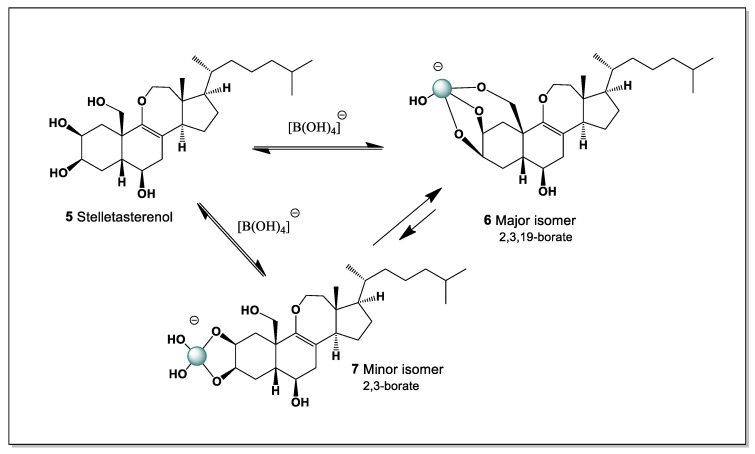
Proposed boron complexes of stelletasterenol. Based on its hydroxylation pattern, stelletasterenol can form two principal boron-complexed isomers under boron-rich aqueous conditions. The predominant species involves cyclic borate ester formation with the vicinal C-2/C-3 diol, while a minor isomer is additionally stabilized through secondary interaction with the angular C-19 hydroxymethyl group, creating an extended multidentate boron-binding environment. These complexes are expected to exist in dynamic equilibrium, with their relative populations governed by local pH, boron availability, and solvent conditions.

**Figure 5:**
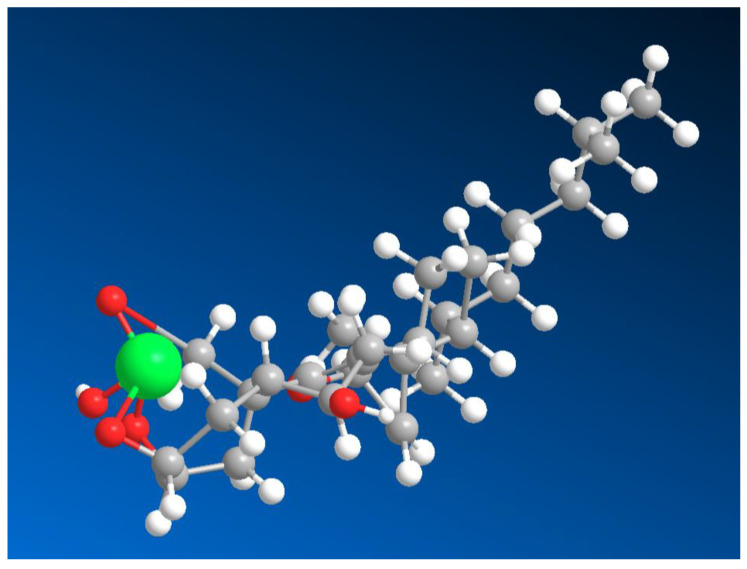
3D structure of the major isomer of the boron complex stelletasterenol, which contains hydroxyl groups at positions 2, 3, and 19 participating in boron coordination. In this structure, the boron atom forms a tetrahedral borate complex with the three oxygen donor atoms of the steroid framework, stabilizing the molecular conformation. The boron atom is shown in green and slightly exaggerated for clarity in order to highlight its coordination environment within the sterol molecule. Such coordination may significantly influence the electronic distribution and conformational rigidity of the steroid backbone.

From a chemical standpoint, stelletasterenol is particularly noteworthy because it bears three strategically positioned hydroxyl groups at C-2, C-3, and C-19. This arrangement creates an ideal boron coordination site, analogous to those observed in ouabain, euryspongiols, and related polyhydroxylated steroids. The vicinal C-2/C-3 diol, combined with the angular C-19 hydroxymethyl group, provides a multidentate binding environment capable of stabilizing tetrahedral borate esters under mildly alkaline or neutral aqueous conditions. Thus, two possible variants of boron complexes can be identified. Specifically, hydroxyl groups C-2 and C-3 form the major isomer, while the minor isomer combines hydroxyl groups at positions C-2, C-3, and C-19 (Figure 4, and 3D structure shown in Figure 5).

Boron coordination at these positions is expected to produce a cyclic borate complex that simultaneously engages the A-ring diol and the C-19 hydroxyl, leading to conformational fixation of the A-ring region. In the case of stelletasterenol, such coordination may also indirectly influence the geometry of the oxygen-bridged 9(11)-seco motif by restricting backbone flexibility. This conformational control could be particularly significant for molecular recognition processes involving PAF receptors, where subtle changes in steric presentation and hydrogen-bonding patterns can markedly affect agonist potency.

The secosteroidal nature of stelletasterenol further enhances its propensity for boron complexation. Ring opening and ether bridge formation increase solvent accessibility and reduce steric shielding of hydroxyl groups compared to fully cyclized steroids. As a result, borate ions can more readily approach and coordinate the polyol region, enabling dynamic and reversible boron binding. Such reversible coordination may modulate biological activity by altering receptor affinity, membrane interactions, or metabolic stability without permanently modifying the steroid core.

From a pharmacological perspective, boron complexation of stelletasterenol could have several consequences: (i) stabilization of reactive hydroxyl groups against enzymatic oxidation; (ii) modulation of polarity and membrane partitioning; and (iii) fine-tuning of PAF agonist activity through conformational locking. These effects parallel observations made for ouabain–boron complexes, where borate coordination induces defined conformational states and alters functional behavior.

In a broader context, stelletasterenol reinforces the emerging concept that marine secosteroids with clustered hydroxyl groups constitute a privileged class of boron-binding natural products. Alongside euryspongiols and other polyhydroxylated secosteroids, stelletasterenol exemplifies how boron coordination chemistry can intersect with lipid mediator signaling, expanding the relevance of boron beyond cardiac glycosides to include inflammatory and platelet-related pathways.

Stelletasterenol highlights the potential of boron coordination as a chemical strategy to modulate the structure and activity of bioactive secosteroids, offering new perspectives for marine natural product chemistry, mechanistic pharmacology, and the rational design of steroid-based modulators of inflammation and thrombosis [93,94,95].

##### MO Study of a Representative Stelletasterenol Molecule (Gaussian DFT)

Molecular orbital calculations were performed on a representative stelletasterenol molecule using DFT methods implemented in Gaussian. Geometry optimization and frontier orbital analysis revealed that the highest occupied molecular orbital is largely localized around the oxygen-containing regions of the molecule, indicating that these atoms act as primary electron-donor sites. The calculated electronic structure suggests that the oxygen atoms provide suitable coordination sites for boron species. Natural bond orbital analysis further indicates significant electron density at these oxygen atoms, supporting the potential formation of borate complexes. Comparison of the free steroid framework with hypothetical borate complexes suggests that boron coordination could stabilize specific conformations of the molecule and alter its electronic properties, thereby potentially influencing its biological activity (see Table 1).

#### 5.1.2. Euryspongiols as Boron-Coordination-Capable Secosteroids

**Figure 6:**
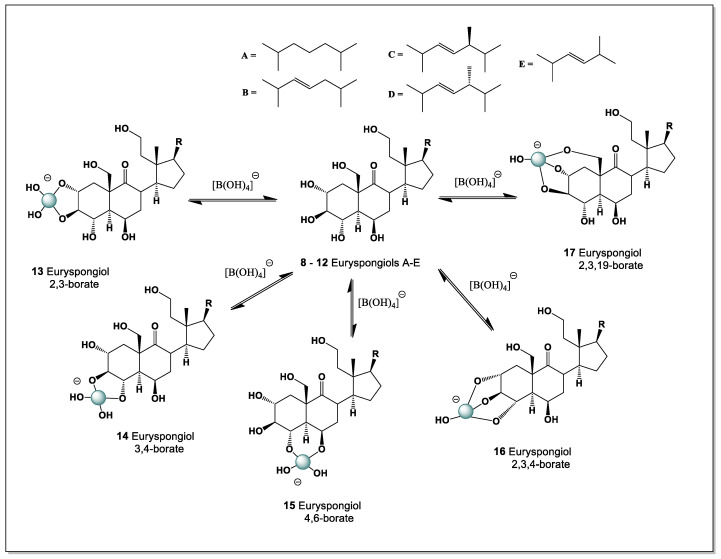
The polyhydroxy steroids known as euryspongiols (**8**–**12**) constitute a distinctive group of marine-derived secosteroids isolated from the sponge *Euryspongia* sp. These compounds possess a highly oxygenated steroidal framework bearing hydroxyl groups at C-2, C-3, C-4, C-6, and C-19. Such a hydroxylation pattern provides several potential sites for boron interaction in boron-rich aqueous environments. While multiple intramolecular borate complexes (**13**–**17**) are theoretically possible, boron coordination is expected to occur preferentially at vicinal diol motifs. Accordingly, borate ester formation involving the C-2/C-3 or C-3/C-4 diol pairs is likely to be favored, whereas alternative binding modes that engage more remote hydroxyl groups may occur only to a limited extent or under specific local conditions.

Euryspongiols (**8**–**12**) represent a distinctive class of marine-derived secosteroids isolated from the sponge *Euryspongia* spp., notable for their antihistamine and anti-inflammatory activities. Structurally, these metabolites are characterized by ring-cleaved (seco) steroid frameworks combined with densely oxygenated polyol motifs, most commonly involving hydroxyl groups at C-2, C-3, and C-19, or alternatively C-2, C-3, and C-4 (see Figure 6). Such arrangements of vicinal and spatially proximal hydroxyl groups are particularly well suited for boron coordination, enabling the formation of cyclic borate esters analogous to those described for ouabain and related cardiac glycosides [14,96].

From a chemical perspective, the presence of cis- or pseudo-*cis* diols at C-2/C-3, together with a third hydroxyl group positioned either on the steroid backbone (C-4) or on the angular methyl-derived C-19 hydroxymethyl group, creates a multidentate binding site capable of stabilizing tetrahedral borate complexes. In alkaline or weakly neutral aqueous environments, borate ions could chelate these hydroxyls, leading to conformational locking of the A-ring or seco-A-ring region. Such coordination is expected to reduce conformational flexibility, alter hydrogen-bonding patterns, and modulate the presentation of polar groups involved in biological recognition.

The secosteroidal nature of euryspongiols further enhances their relevance to boron chemistry. Ring cleavage increases molecular flexibility and solvent accessibility of hydroxyl groups, facilitating borate ester formation compared to rigid, fully cyclized steroids. This structural openness may also allow dynamic boron coordination, enabling reversible complex formation that could respond to changes in pH, ionic strength, or local metabolite concentrations—features consistent with bioactive regulation rather than permanent covalent modification.

From a pharmacological standpoint, boron complexation of euryspongiols could plausibly influence their antihistamine activity by modifying interactions with histamine receptors, membrane lipids, or inflammatory mediators. Borate coordination may also enhance chemical stability, protect reactive hydroxyl groups from metabolic oxidation, or subtly alter lipophilicity and membrane permeability. These effects parallel observations made for ouabain–boron complexes, where boron binding induces defined conformational states and measurable changes in chemical reactivity.

Euryspongiols expand the scope of boron-binding steroids beyond cardiac glycosides, demonstrating that marine secosteroids with clustered hydroxyl groups represent an additional, underexplored class of boron-coordination-capable natural products. Their unique combination of polyol-rich scaffolds, seco-steroid topology, and bioactivity supports the broader concept that boron coordination may act as a general modulatory strategy in steroid chemistry, with implications for drug design, chemical ecology, and the development of novel steroid-based therapeutics.

The polyhydroxy steroids designated as euryspongiols (**8**–**12**) represent a distinctive class of marine secosteroids isolated from the sponge *Euryspongia* sp., characterized by an exceptionally dense array of hydroxyl groups at C-2, C-3, C-4, C-6, and C-19. This structural organization creates a versatile boron-binding platform capable of generating multiple regioisomeric borate complexes in boron-rich marine environments. In principle, up to five different intramolecular boron complexes (**13**–**17**) may be formed, reflecting alternative combinations of hydroxyl groups engaged in coordination. Among these, tetrahedral borate esters involving the vicinal C-2/C-3 (**13**) or C-3/C-4 (**14**) diol units are expected to be thermodynamically preferred and therefore dominant, while additional, less populated complexes incorporating distal hydroxyl groups such as C-6 or C-19 may contribute to a dynamic speciation ensemble.

#### 5.1.3. Bienmasterol from Marine Sponge *Bienma*

Bienmasterol (**18**), a marine steroid isolated from the sponge *Bienma* sp. collected near Okinawa Island (Japan), represents an unusual example of sulfur-related natural products from marine organisms [97]. This compound exhibits cytotoxic activity against tumor cell lines, including L-1210 leukemia cells and KB carcinoma cells. Structurally, bienmasterol is distinguished by the presence of a 22,25-diene fragment in the sterol side chain, a structural motif that is relatively rare among naturally occurring steroids. The occurrence of such modified sterol structures in marine sponges reflects the remarkable biosynthetic diversity of marine invertebrates, which often produce structurally unique metabolites with potent biological activities. These findings highlight marine sponges as important sources of pharmacologically interesting sterol derivatives with potential applications in anticancer drug discovery.

**Figure 7:**
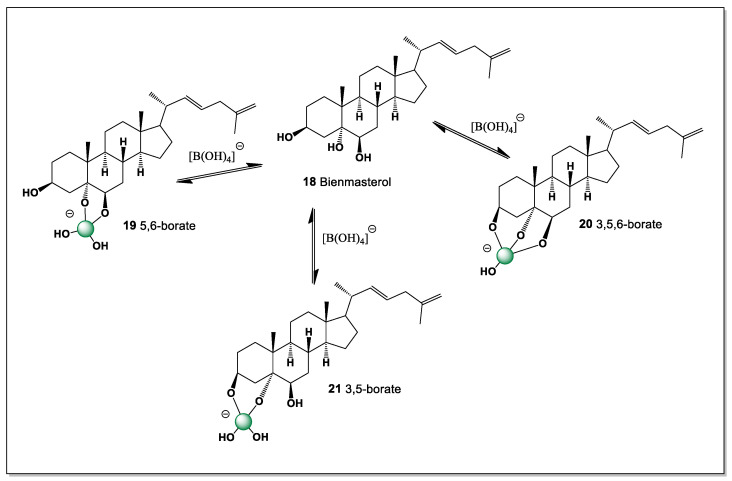
The sea sponge *Bienma* sp. contains the cytotoxic bienmasterol, which bears hydroxyl groups at positions 3, 5, and 6 and exists as three isomeric forms, with the boron complex being the dominant species (**19**). The formation of this boron complex is attributed to the presence of vicinal hydroxyl groups that enable stable borate ester formation. This structural feature may contribute to the observed cytotoxic activity by modulating molecular conformation and biological interactions.

This steroid, with hydroxyl groups at positions 3, 5, and 6, can have only three isomers of boron complexes (**19**–**21**). The main isomer is the one with a 1,2-diol group, i.e., 5,6-borate (see Figure 7).

This preference reflects the favorable chelation of boron with the vicinal 5,6-diol, which forms a stable five-membered borate ring and minimizes steric strain within the steroid nucleus. The dominance of the 5,6-borate thus provides indirect support for the assigned stereochemistry of the A/B ring system and highlights how subtle side-chain unsaturation can coexist with selective complexation behavior in marine steroids.

#### 5.1.4. Contignasterol from the Marine Sponge *Petrosia contignata*

Contignasterol (**22**) is a highly oxidized marine steroid with a unique structure and pronounced biological activity. It was first isolated in 1992 from the marine sponge *Petrosia contignata*, collected in the waters of Papua New Guinea [98]. It is notable for the presence of an unusual 14β-hydrogen configuration, making it the first naturally occurring steroid discovered with this stereochemical arrangement. Its molecule also contains a cyclic hemiacetal group in the side chain, a rare feature among marine sterols that contributes to its distinctive chemical reactivity. The biological activity of contignasterol is expressed through powerful anti-inflammatory and antiallergic effects, including strong suppression of histamine release from mast cells and reduction of airway tissue swelling during allergic responses.

**Figure 8:**
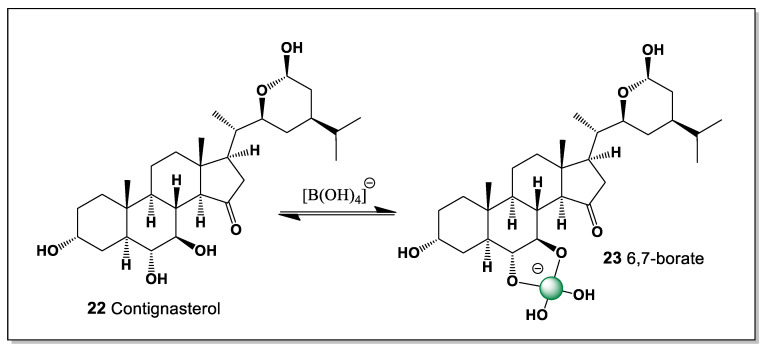
Contignasterol was isolated from an extract of the marine sponge *Petrosia contignata* and occurs as a single isomer with hydroxyl substitution at positions 6 and 7 (**23**). The restricted isomerism suggests a more rigid steroidal framework compared to related sponge-derived sterols. Such structural simplicity may influence its interaction with boron species and could affect both complex formation and biological activity.

Structurally, contignasterol possesses adjacent hydroxyl groups at positions C-6 and C-7, which provide a suitable cis-diol motif capable of interacting with boron species. As a result, it can theoretically form a single boron-coordinated complex (**23**) through chelation with boric acid or borate anions (Figure 8). The formation of such a boron complex may influence the conformation, polarity, and intermolecular interactions of the steroid molecule. This interaction could potentially modify the compound’s membrane affinity or biological activity, particularly in aqueous environments where borate species are present. From a chemical perspective, the presence of the 6,7-diol system makes contignasterol an interesting candidate for studying boron–diol coordination in marine natural products. These structural features highlight the potential role of boron coordination chemistry in modulating the biological properties of complex marine sterols.

#### 5.1.5. Xestobergsterol B Derived from *Xestospongia* and *Ircinia* Sponges

Xestobergsterol B (**24**), containing five carbocycles with cis-linked rings C and D, was found in *Xestospongia bergquistia* and *Ircinia* sp. [99,100,101]. The presence of an additional ring in these steroids is presumably due to intramolecular aldol condensation of an as-yet-unisolated 23-keto derivative. It exhibits cytotoxic activity and inhibits histamine release from mast cells induced by anti-IgE when administered at low doses. Therefore, this compound is of considerable interest as a potential active ingredient in new antihistamines. Its activity is closely linked to potent inhibition of phosphatidylinositol phospholipase C.

**Figure 9:**
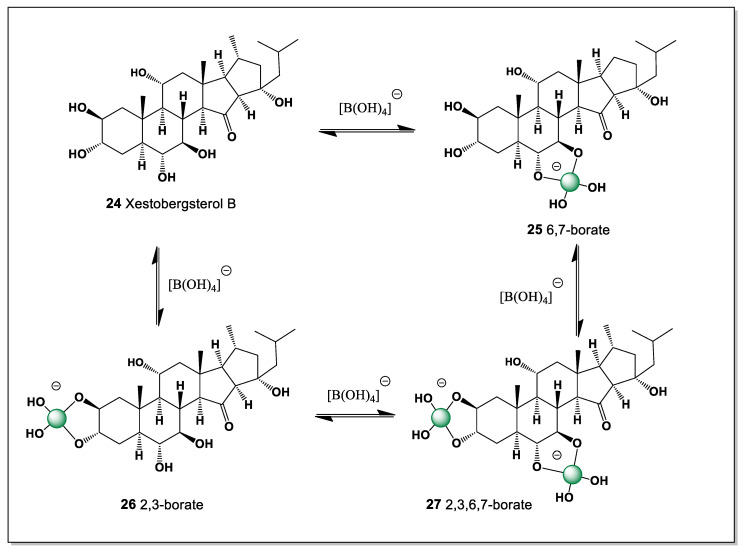
Xestobergsterol B (**24**), a steroid identified in extracts of the sea sponges *Xestospongia bergquistia* and *Ircinia*, forms three distinct boron complexes (**25**, **26**, and **27**). The presence of multiple hydroxyl functionalities enables alternative boron coordination modes, giving rise to structurally distinct complexes. Such variability in boron binding may influence the compound’s conformational flexibility and stability in biological environments. These boron complexes highlight the chemical versatility of sponge-derived steroids as scaffolds for boron-based bioactive molecules.

Based on the theoretical assumptions of the structure of this steroid, which has cis-1,2-diol groups at positions 2 and 3, as well as at positions 6 and 7, it is difficult to determine which of these two isomers has superior stability, but both form boron complexes (**25** and **26**) (Figure 9). This depends on the conformation of this steroid; however, both of these boron complexes are equally likely to form a mixed boron complex (**27**).

The formation of the mixed boron complex can be rationalized by the conformational flexibility of the polycyclic framework, which allows simultaneous or competitive coordination of boron to both *cis*-1,2-diol motifs. This behavior underscores the comparable chelating ability of the 2,3- and 6,7-diol systems and further supports the proposed stereochemical arrangement of the rings, as neither diol pair is strongly disfavored on steric or electronic grounds.

#### 5.1.6. Xestokerol A and Aragusterol C Derived from *Xestospongia* Sponge

**Figure 10:**
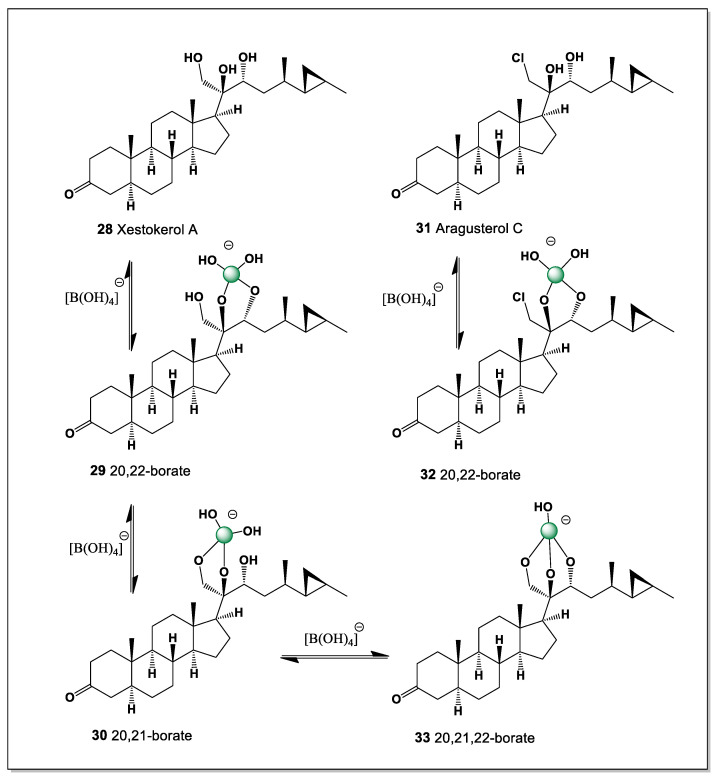
Two steroids, xestokerol A (**28**) and aragusterol C (**31**), produced by the sponge *Xestospongia*, are shown. Xestokerol A exists in three isomeric forms (**29**, **30**, and **33**), whereas aragusterol C occurs as a single isomer (**32**). The multiple isomers of xestokerol A reflect greater flexibility in functional group orientation, which may facilitate alternative modes of boron complexation. In contrast, the limited isomerism of aragusterol C suggests a more constrained steroidal framework. These differences highlight how subtle structural variations among sponge-derived steroids can influence their capacity to form boron-containing complexes and potentially modulate biological activity.

Two remarkable lipid hormones, 26,27-cyclosterols, xestokerol A and aragusterol C, were isolated from the Okinawan marine sponge of the genus *Xestospongia* [102,103]. Xestokerol A (**28**) and aragusterol C (**31**) belong to the sponge’s polyhydroxysteroids (Figure 10). Aragusterols and related compounds have unusual side chains containing cyclopropane moieties, while aragusterol C contains a chlorine atom in the side chain, a unique structural feature of natural steroids.

Xestokerol A contains four hydroxyl groups, three at positions 20, 21, and 22, while aragusterol C has the hydroxyl group at position 21 replaced by a chlorine atom. This is a rare combination of hydroxyl groups in steroids. Thus, xestokerol A has the ability to form three isomers of boron complexes (**29**, **30**, and **33**), as shown in Figure 9, while aragustrol C, unlike xestokerol A, has only one isomer (**32**).

Qualitative molecular-orbital comparison of stelletasterenol (**5**) and xestokerol A (**28**) and their proposed boron complexes is shown in Table 2.

The presence of clustered hydroxyl groups at C20–C22 in xestokerol A creates an uncommon, side-chain-localized chelation domain that is well suited for boron coordination and supports multiple borate geometries. In contrast, substitution of the C21 hydroxyl group by chlorine in aragusterol C disrupts potential diol or triol motifs, drastically reducing the number of viable boron-binding arrangements to a single isomer. This comparison illustrates how even a single heteroatom substitution in the steroid side chain can profoundly alter boron complexation behavior. More broadly, these 26,27-cyclosterols from sponges of the genus *Xestospongia* underscore the sensitivity of boron–steroid interactions to subtle structural variations in polyhydroxylated marine lipids.

#### 5.1.7. Three Pregnane Derivatives Derived from *Strongylophora* Sponge

**Figure 11:**
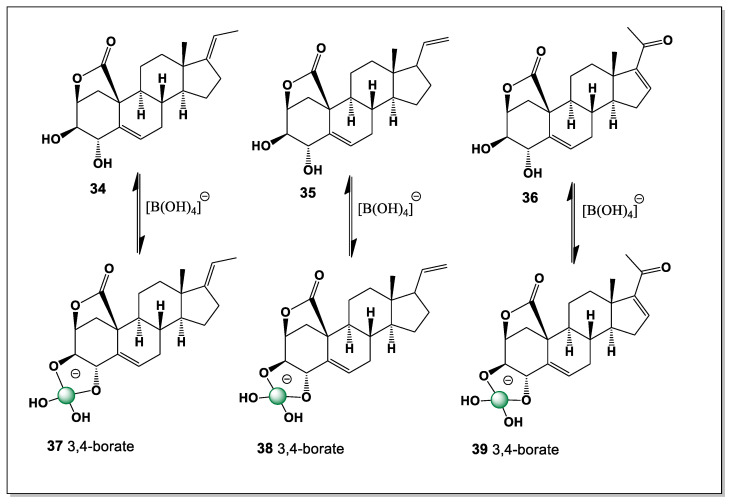
Three pregnane derivatives were obtained from the sponge *Strongylophora*, and all three pregnanes form a single boron complex (borates 3 and 4). The uniform formation of one boron complex suggests a conserved arrangement of hydroxyl groups across these pregnane structures. Such structural consistency may favor selective and stable borate ester formation. This behavior further supports pregnane-type steroids from marine sponges as reliable scaffolds for boron incorporation in bioactive molecules.

*Strongylophora* is a subgenus (previously considered a separate genus) of marine sponges belonging to the family Petrosiidae and the genus *Petrosia*. Members of the class Demospongiae and the order Haplosclerida, these sponges are a source of unique metabolites, such as strongylophorines—meroditerpenoids with antimicrobial, cytotoxic, and neuroprotective activity—and strongylodiols—long-chain acetylenic alcohols. The sponge is found in tropical waters of the Indo-Pacific region, including Okinawa, the Philippines, and Thailand. Three pregnanes, 3,4-dihydroxypregna-5,17-diene-10,2-carbolactone (**34**), 3,4-dihydroxypregna-5,20-diene-10,2-carbolactone (**35**), and 3,4-dihydroxypregna-5,15-dien-20-one-10,2-carbolactone (**36**), were isolated from this sponge, *Strongylophora* sp. [104]. All isolated steroids contained only two hydroxyl groups (3 and 4), so all steroids can form only one isomer of boron complexes, such as (**37**), (**38**), and (**39**) (Figure 11).

The limited hydroxylation pattern of these pregnane derivatives imposes a strong conformational restriction on boron coordination, permitting only a single, well-defined chelation mode. In each case, the 3,4-diol arrangement provides the sole viable donor set capable of forming a stable boron–oxygen complex, while the lactone functionality does not significantly participate in boron binding. This simplicity contrasts with the highly polyhydroxylated steroids found in other marine invertebrates and highlights a different strategy of structural preorganization. The findings from *Strongylophora* sp. emphasize that even minimally hydroxylated marine steroids can engage in boron complexation when the diol geometry is optimal.

#### 5.1.8. An Unusual 9,11-Secosteroid, Blancasterol Derived from *Pleraplysilla* Sponge

**Figure 12:**
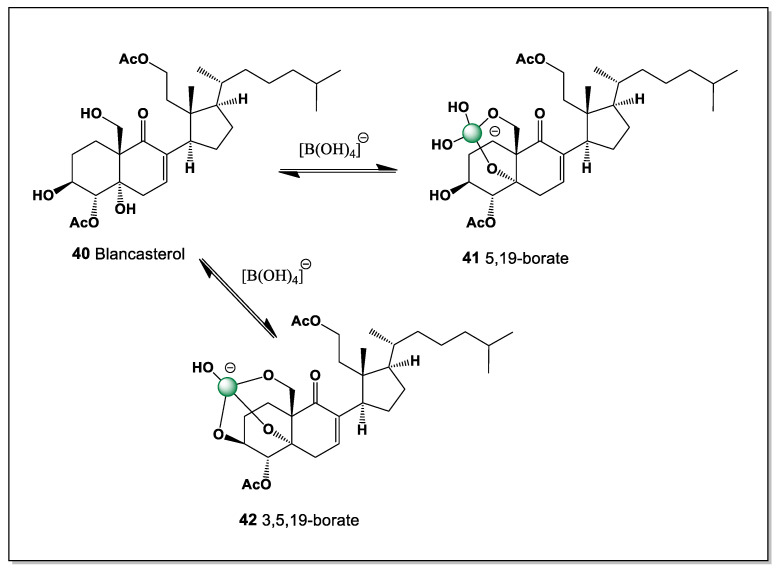
An unusual 9,11-secosteroid, blancasterol, was discovered in the sponge *Pleraplysilla* and occurs as two isomeric forms: one bearing hydroxyl groups at positions 5 and 19 (**41**) and another substituted at positions 3, 5, and 19 (**42**). The presence of multiple hydroxyl groups provides potential coordination sites for boron, enabling the formation of distinct boron complexes. The secosteroidal framework further enhances structural flexibility, which may influence boron binding geometry. These features underscore the chemical uniqueness of blancasterol as a marine-derived scaffold for boron-containing steroidal derivatives.

*Pleraplysilla* is a genus of marine sponges in the family Aplysillidae (order Dictyoceratida), known for producing unique furanosesquiterpenes. These sponges are a source of bioactive compounds such as pleraplysillin, pleraplysillin-2, and dehydrodendrolasin, which have interesting chemical structures, such as terminal (beta)-monosubstituted furan rings. They are of interest to marine pharmacognosy due to the presence of specific chemical components. An unusual 9,11-secosteroid called blancasterol (**40**), a cytotoxic diacetate from the Northern Pacific sponge *Pleraplysilla* sp., inhibits the growth of various tumor cells, including drug-resistant cell cultures of human mammary carcinoma MCF-7 [105]. This secosteroid contained three hydroxyl groups at positions 3, 5, and 19. Theoretically, it could have two isomers: one at 5 and 19 (**41**), and another at 3, 5, and 19 (**42**) (Figure 12).

The presence of only three hydroxyl groups in blancasterol imposes strict limitations on boron coordination, confining complex formation to a small number of stereochemically feasible arrangements. The 5,19-diol motif is expected to provide the most favorable geometry for boron chelation, whereas simultaneous involvement of the 3-, 5-, and 19-hydroxyl groups would require greater conformational distortion of the secosteroid framework. The 9,11-seco structure further influences donor–donor distances, differentiating blancasterol from conventional tetracyclic steroids in its boron-binding behavior. These features underscore how oxidative ring cleavage in marine secosteroids from *Pleraplysilla* can generate distinct coordination environments that modulate both chemical reactivity and biological activity.

#### 5.1.9. A Unique Steroid Containing a 5,19-Cycloergostane Skeleton

**Figure 13:**
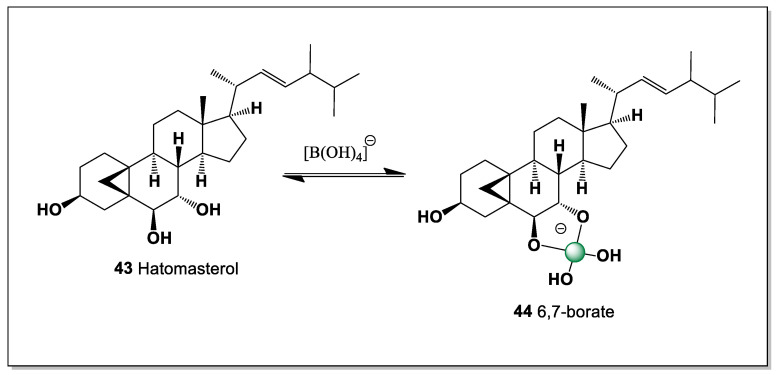
A unique steroid containing a 5,19-cycloergostane skeleton (**43**) was isolated from a marine sponge *Stylissa*. The compound, named hatomasterol (**43**), bears three hydroxyl groups at positions 3, 6, and 7 and forms only a single boron complex isomer (6 and 7). The constrained 5,19-cyclized framework likely limits conformational flexibility, favoring selective boron coordination. This structural rigidity may contribute to the stability and defined geometry of the resulting boron complex.

*Stylissa* is a genus of marine sponges in the family Scopalinidae, found primarily in tropical waters of the Indo-Pacific region and the Red Sea [106]. They are widely known in the scientific community as a rich source of unique bioactive compounds with potential for drug development. They typically inhabit shallow waters (3 to 23 m), attaching to hard substrates such as rocks or coral reefs. The genus *Stylissa* is of great interest to pharmacologists due to the active secondary metabolites they contain. A unique steroid containing a 5,19-cycloergostane skeleton, (3,5,6,7,22*E*,24ς)-5,19-cycloergost- 22-ene-3,6,7-triol, named hatomasterol (**43**, see Figure 13), was found in extracts of the Okinawan sponge *Stylissa* sp., and this compound demonstrated cytotoxicity against HeLa cells in vitro [107]. The steroid contains three hydroxyl groups (3, 6, and 7), so it can form only one isomer of boron complexes (6 and 7) (**44**).

The unusual 5,19-cycloergostane framework of hatomasterol imposes a highly constrained three-dimensional geometry that limits the number of feasible boron coordination modes. With only the 6,7-diol providing a suitably oriented donor pair, boron complexation is restricted to a single, well-defined isomer. This structural simplicity contrasts with the more flexible polyhydroxysteroids found in other marine sponges, yet still permits effective boron binding due to precise stereochemical preorganization. The example of hatomasterol from *Stylissa* highlights how rigid, unconventional steroid skeletons can support selective boron–oxygen interactions while retaining significant cytotoxic activity.

### 5.2. Polyhydroxysteroids Derived from Echinodermata

Echinodermata is a phylum of exclusively marine, benthic animals, including starfish, sea urchins, sea cucumbers, brittle stars, and sea lilies. The name derives from Greek words meaning “spiny skin,” reflecting characteristic features of the group. Adult echinoderms typically exhibit five-ray radial symmetry, whereas their larvae are bilaterally symmetrical. A notable biological trait of this phylum is their pronounced regenerative capacity; for example, some sea cucumbers can regenerate entire internal organs after evisceration as a defensive strategy.

Marine steroids released by benthic invertebrates or their associated microorganisms may accumulate in surrounding sediments and on the seafloor, where organic matter and mineral surfaces facilitate their retention. In these settings, elevated and geochemically buffered boron levels provide conditions under which reversible boron–diol interactions with polyhydroxylated steroids are chemically plausible and potentially recurrent [107,108,109].

Echinoderms are also notable producers of structurally diverse steroids, many of which are highly oxygenated and play important roles in chemical defense and ecological interactions. Given that boron is abundant in marine sediments and on the seafloor, the close association of echinoderms with these environments may facilitate the formation and accumulation of boron–steroid complexes through interactions between sediment-derived boron species and hydroxyl-rich steroidal metabolites [110,111,112,113].

#### 5.2.1. The Starfish *Archaster typicus*

*Archaster typicus*, also known as the sand sifting starfish, is a species of echinoderm common in the shallow waters of the Indo-Pacific region [114,115]. The starfish is adapted to life on sandy bottoms, burying itself in the sand during high tide and moving across the surface during low tide. It feeds on detritus, organic remains and small invertebrates, sifting through the top layer of soil, which may contain increased levels of boron from boron-containing minerals.

Two (22*E*, 24*R*, 25*R*)-24-methyl-5α-cholest-22-ene-3β, 4β, 5, 6α, 8, 14, 15α, 25, 26-nonaol (**45**) and (22*E*, 24*S*)-24-methyl–5α-cholest-22-ene-3β, 4β, 5, 6α, 8, 14, 15α, 25, 28-nonaol (**46**), minor marine polyhydroxysteroids were isolated from the starfish *Archaster typicus* [116].

These highly oxygenated steroids are characterized by an unusually dense array of hydroxyl groups distributed over both the steroid nucleus and the side chain, resulting in a rigid yet strongly hydrophilic molecular architecture. Such extensive polyhydroxylation creates favorable spatial arrangements for multidentate coordination, making these compounds particularly well suited for the formation of stable boron–oxygen complexes. The occurrence of these minor polyhydroxysteroids in *Archaster typicus* further supports the hypothesis that marine steroids with high hydroxyl density may act as natural ligands for boron, contributing to the emergence of marine boronosteroids.

**Figure 14:**
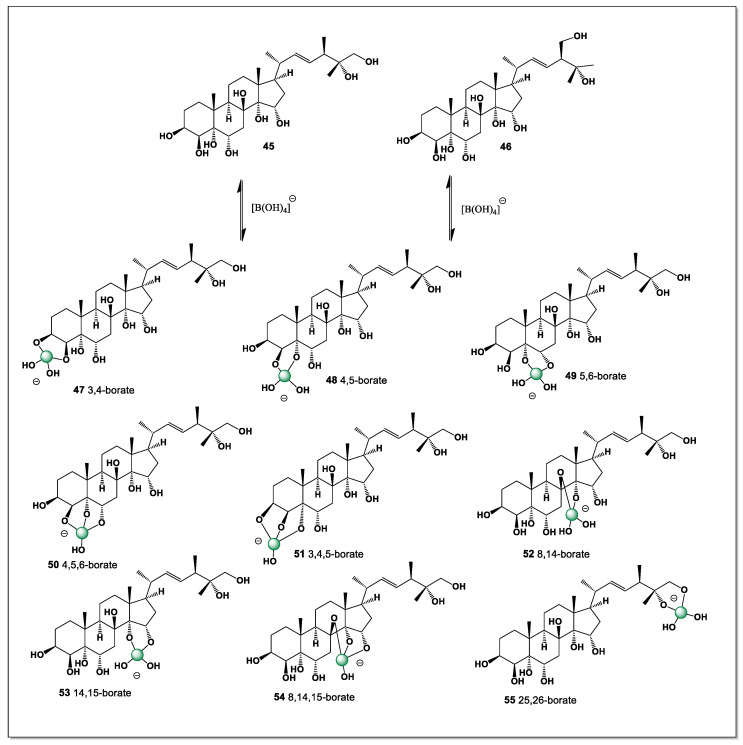
The starfish *Archaster typicus* produces two steroids that generate eight boron complex isomers (**47**–**54**), while compound **45** forms a ninth isomer (**55**) derived from hydroxyl groups at carbon atoms 25 and 26. The additional boron complex reflects the presence of a flexible side chain bearing suitably positioned diol functionalities. This expanded isomerism highlights how subtle differences in hydroxyl group placement can markedly increase boron coordination diversity in marine-derived steroids.

This is perhaps a very rare case of steroids containing nine hydroxyl groups found in marine invertebrates, specifically the starfish *Archaster typicus*. Both isolated steroids could theoretically contain eight isomers of boron complexes (**47**–**54**), but **45** contains a ninth isomer (**55**), formed from the hydroxyl groups at carbon atoms 25 and 26. The dominant isomers could be **48**, **49**, **50**, **52**, **53**, and **55**, as shown in Figure 14.

This additional coordination site further increases the structural complexity and conformational diversity of compound **28** compared with related polyhydroxylated steroids. Such extensive hydroxylation may significantly influence both the stability of the boron complexes and their spectroscopic behavior, complicating unambiguous structural assignment. Moreover, the presence of multiple dominant isomers suggests a dynamic equilibrium in solution, which could be relevant to understanding the chemical reactivity and potential biological roles of these unusual marine steroids.

#### 5.2.2. The Garlic Marine Star *Dermasterias imbricata*

*Dermasterias imbricata*, commonly known as the leather sea star or garlic sea star, is a species of echinoderm native to the Pacific coast of North America. It typically has five broad arms and a broad central disk. The upper surface is smooth and slippery to the touch, resembling wet leather, due to mucus secretion and the absence of spines. It often has a mottled, blue-gray pattern with reddish-orange or brown spots. A characteristic feature is a pungent odor reminiscent of garlic, sulfur, or burnt gunpowder, especially noticeable when the starfish is removed from the water [117].

**Figure 15:**
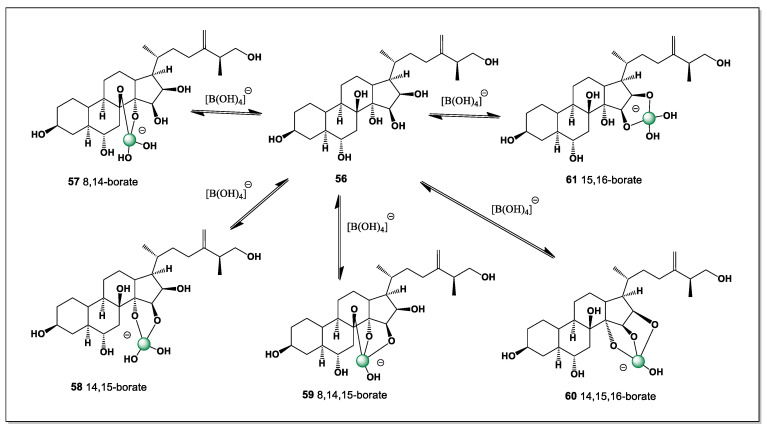
The Californian starfish *Dermasteria imbricata* produces polyhydroxylated sterols, including a compound bearing seven hydroxyl groups (**56**) that can form five distinct boron complexes. The high degree of hydroxylation provides multiple potential coordination sites, enabling diverse boron-binding patterns. Such extensive boron complexation underscores the remarkable chemodiversity of starfish sterols and their potential as versatile boron-containing scaffolds.

The *Dermasteria imbricata* sea star from the Gulf of California contained six polyhydroxylated sterols, one of which contained seven hydroxyl groups (**56**), which can contain five isomers of boron complexes, as shown in Figure 15. Hydroxyl groups are located at positions 3, 6, 8, 14, 15, 16, and 26 [118].

The exceptionally high degree of hydroxylation in these sterols provides multiple oxygen donor sites arranged on a rigid 5α-steroidal framework, creating favorable geometries for boron coordination. As a result, a single heptahydroxylated sterol can give rise to several distinct boron complexes (**57**–**61**), differing in chelation mode and stereochemical arrangement, thereby accounting for the four observed boron isomers. This finding highlights the capacity of polyhydroxylated echinoderm sterols to act as versatile natural ligands for boron in marine environments, particularly in sediment-rich regions such as the Gulf of California.

The predominance of boron complex isomers **57**, **58**, **59**, and **61** suggests that only a limited number of coordination geometries are energetically favored, despite the large number of potential hydroxyl donor sites available in the parent sterol. These dominant isomers likely correspond to chelation modes that best accommodate boron’s preferred trigonal or tetrahedral coordination while minimizing steric strain imposed by the rigid steroid nucleus.

#### 5.2.3. The Starfish *Patiria pectinifera*

*Patiria pectinifera* (formerly known as *Asterina pectinifera*) is a species of starfish found in the Northern Pacific Ocean, known for its use as a model organism in developmental biology and as a potential source of useful biomedical compounds [119].

The starfish *Asterina pectinifera* (order Spinulosa), feeding on oyster, abalone, and other echinoderms was widely distributed in North Pacific Ocean. Extracts from *A. pectinifera* are being researched for their potential use in pharmaceutical and cosmetic products, as they contain beneficial compounds like collagen peptides and astaxanthin-enriched carotenoids [120].

**Figure 16:**
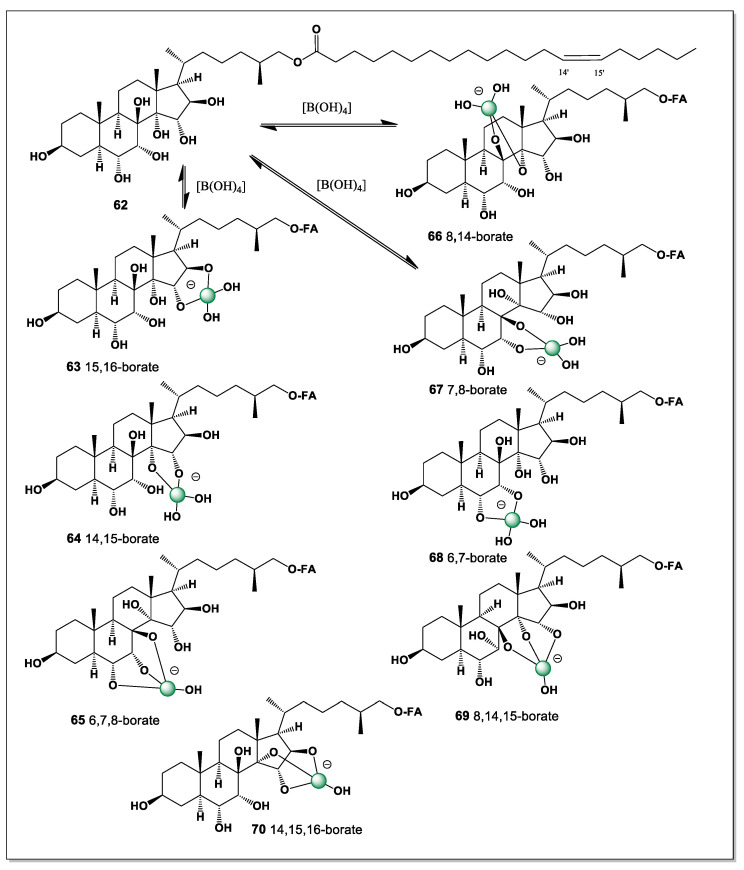
*Patiria pectinifera* (formerly known as *Asterina pectinifera*), a Pacific starfish, contains a series of unusual steroids. The steroid (**62**) can form eight possible boron complex isomers (**63**–**70**). This extensive isomerism arises from the presence of multiple hydroxyl groups arranged in different spatial orientations. Such variability enables alternative boron coordination modes, greatly expanding structural diversity. These features emphasize the potential of starfish-derived steroids as rich sources of multifunctional boron-containing natural products.

The search for cytotoxic compounds from the starfish *A. pectinifera* was successful and a polyhydroxysterol ester (25*S*)-5-acholestan-3β,6α,7α,8,15α,16β- hexahydroxyl-26-O-14-zeicosenoate (**62**) was isolated. The hydroxyl groups are at positions 3, 6, 7, 8, 15, 16, and 26. Thus, this steroid has eight possible isomers of boron complexes (**63**–**70**), such as 6 and 7, 7 and 8, 6, 7 and 8, 14 and 15, 8 and 14, 8, 14, 15, 14, 15, and 16, and 15 and 16 (see Figure 16).

The presence of multiple vicinal and spatially proximate hydroxyl groups enables several distinct chelation motifs, each giving rise to a different boron complex isomer. Coordination involving the A/B ring hydroxyls (6–7 and 7–8) is likely favored by the rigid 5α-steroidal framework, whereas chelation at positions 15 and 16 reflects the greater conformational adaptability of the C/D ring region. These alternative boron-binding modes underscore how the distribution of hydroxyl groups across the steroid nucleus and side chain governs both the diversity and stability of boron–steroid complexes in marine echinoderms.

#### 5.2.4. Pacific Starfish *Asterina pectinifera*

**Figure 17:**
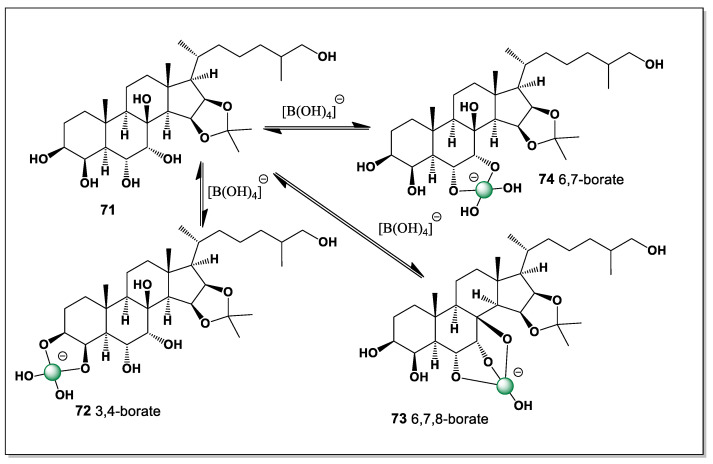
The steroid (**71**) isolated from the starfish *Asterina pectinifera* bears six hydroxyl groups at positions 3, 4, 6, 7, 8, and 26 and has the potential to form three boron complex isomers (**72**–**74**). The defined yet flexible arrangement of these hydroxyl groups allows selective boron coordination at distinct diol sites. This limited number of boron isomers suggests a balance between structural rigidity and functional versatility in this polyhydroxylated steroid.

The rare polyhydroxyl sterol 15β,16β-isopropylidenedioxy-5α-cholestane -3β,4β,6α,7α,8,26-hexaol (**71**) was detected in the whole-body extract of a common Pacific starfish *Asterina pectinifera* [121]. This steroid has six hydroxyl groups at positions 3, 4, 6, 7, 8, and 26 and has the potential to form three isomers of boron complexes (**72**–**74**, Figure 17). The isomers of the boron complexes are shown in Figure 16. It can be predicted that isomer **73** will be the dominant one.

The preference for isomer **73** can be rationalized by the favorable spatial alignment of vicinal hydroxyl groups within the rigid 5α-steroidal framework, which allows optimal O–B–O chelation with minimal steric strain. In contrast, the alternative coordination modes leading to isomers **72** and **74** likely involve less favorable hydroxyl orientations or increased conformational distortion of the steroid nucleus. The presence of the 15β,16β-isopropylidenedioxy moiety further restricts local flexibility, biasing boron coordination toward a single dominant geometry. These considerations emphasize the strong influence of stereochemical preorganization on the speciation of boron complexes formed by polyhydroxylated marine sterols.

#### 5.2.5. Deep-Sea Starfish *Ctenodiscus crispatus*

**Figure 18:**
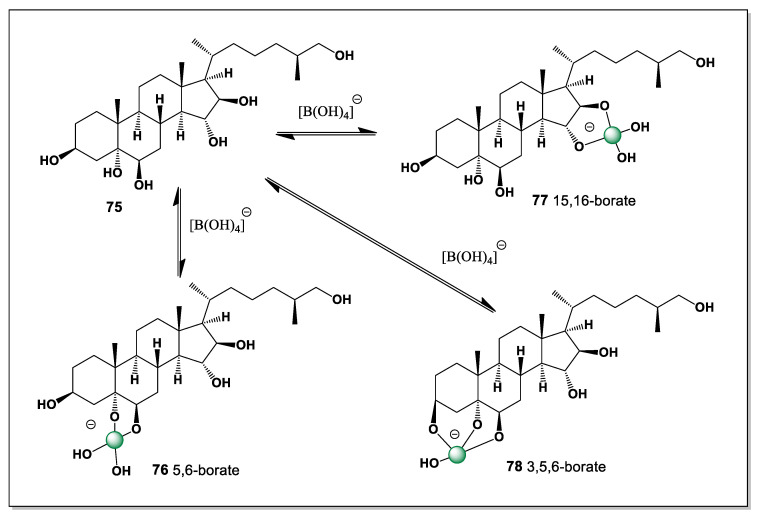
The deep-sea starfish *Ctenodiscus crispatus* contains a steroid bearing six hydroxyl groups at positions 3, 5, 6, 15, 16, and 26 that is capable of forming only three boron complex isomers. The restricted number of boron complexes indicates selective coordination despite the high degree of hydroxylation. This selectivity likely arises from steric constraints and the spatial orientation of the hydroxyl groups within the steroidal framework. Such controlled boron complexation highlights the influence of molecular topology on boron-binding behavior in deep-sea starfish steroids.

*Ctenodiscus crispatus*, commonly known as the mud star or cookie-cutter sea star, is a species of deep-sea starfish in the family Ctenodiscidae. It inhabits arctic and boreal (northern temperate) waters at depths ranging from 400 to 2000 m. It prefers soft, muddy, or sandy sediments. It is a nonselective detritivore. It burrows into the mud and ingests sediment, digesting organic particles and small organisms such as foraminifera and small mollusks. Unique steroids are isolated from the tissues of *Ctenodiscus crispatus*, which are being studied for their cytotoxic effects on cancer cells (e.g., hepatocellular carcinoma). The species is widely distributed in the Arctic Ocean, the North Atlantic (from the Arctic to the coasts of North Carolina and Panama), and the North Pacific Ocean (the Bering Sea, the Oregon coast, and the Sea of Japan) [122,123].

(25S)-5α-cholestane3β,5,6β,15α,16β,26-hexaol (**75**, Figure 18), showing cytotoxic activity against hepatocellular carcinoma cells HepG2in MTTassay, was found in the Mudstar extract of *Ctenodiscus crispatus*. The steroid contains six hydroxyl groups at positions 3, 5, 6, 15, 16, and 26, and is capable of forming only three isomers of boron complexes (**76**–**78**) with the dominant isomers 5 and 6 (**76**), and the second 15 and 16 (**77**) [124].

The deep-sea habitat of *Ctenodiscus crispatus*, characterized by low temperatures, high hydrostatic pressure, and sediment-rich environments, is known to promote the accumulation of unusual secondary metabolites with enhanced chemical stability. As a sediment-ingesting detritivore, this species is continuously exposed to inorganic elements, including boron, present in pore waters and seafloor deposits, creating favorable conditions for in vivo boron–steroid interactions. The restricted number of boron complex isomers formed by compound **75** reflects both the rigidity of the 5α-steroidal framework and the constrained spatial arrangement of its hydroxyl groups. These general features highlight deep-sea echinoderms as particularly promising sources of structurally preorganized polyhydroxysteroids capable of selective boron coordination and biologically relevant activity.

#### 5.2.6. Gray Sea Star *Luidia clathrata*

**Figure 19:**
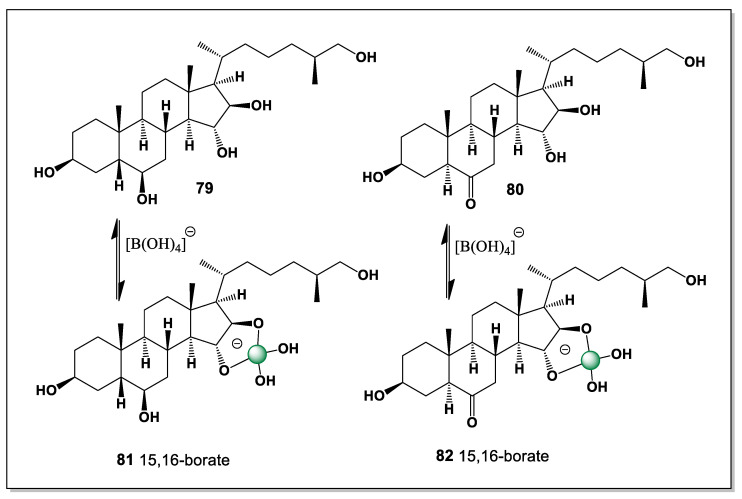
The starfish *Luidia clathrata* from the western Atlantic Ocean contains two steroids (**79** and **80**) bearing five hydroxyl groups at positions 3, 6, 15, 16, and 26 and four hydroxyl groups at positions 3, 15, 16, and 26, respectively, yet each forms only a single boron complex isomer. The presence of only one boron isomer despite multiple hydroxyl groups suggests a highly constrained coordination environment. Steric effects and fixed spatial orientation of the hydroxyl functionalities likely favor a single, thermodynamically stable boron-binding mode. This observation underscores that the number of hydroxyl groups alone does not dictate boron complex diversity in starfish-derived steroids.

*Luidia clathrata* (commonly known as the striped or gray sea star) is a tropical sea star native to the western Atlantic Ocean. It is found from Virginia (USA) to Brazil, including the Gulf of Mexico and the Caribbean Sea. It prefers soft sandy or muddy bottoms in shallow waters (up to 40 m), where it can burrow into sediment. It has a high ability to regenerate lost rays, which may be influenced by steroids [125,126,127]. Two steroids (**79** and **80**, Figure 19) containing five (3, 6, 15, 16, and 26) and four (3, 15, 16, and 26) hydroxyl groups have been isolated from this starfish. The number and conformation of these groups suggests that they can only have one isomer of boron complexes (15 and 16) (**81** and **82**, respectively).

The restriction to a single boron complex isomer can be attributed to the spatial isolation of the hydroxyl groups on the steroid nucleus and side chain, which limits the number of viable chelation pairs. In both steroids, the 15,16-diol located in the C/D ring region provides the only suitably oriented donor set capable of forming a stable boron–oxygen chelate. This conformational constraint reinforces the concept that, in marine starfish steroids, boron complexation is governed more by stereochemical preorganization than by the absolute number of hydroxyl groups present.

#### 5.2.7. The Deep-Sea Starfish *Henricia leviuscula*

The deep-sea starfish *Henricia leviuscula*, better known as the Pacific blood star, is a species of sea star native to the Pacific coast of North America. It is found from the Aleutian Islands (Alaska) to Baja California (Mexico), as well as off the coast of Japan.

**Figure 20:**
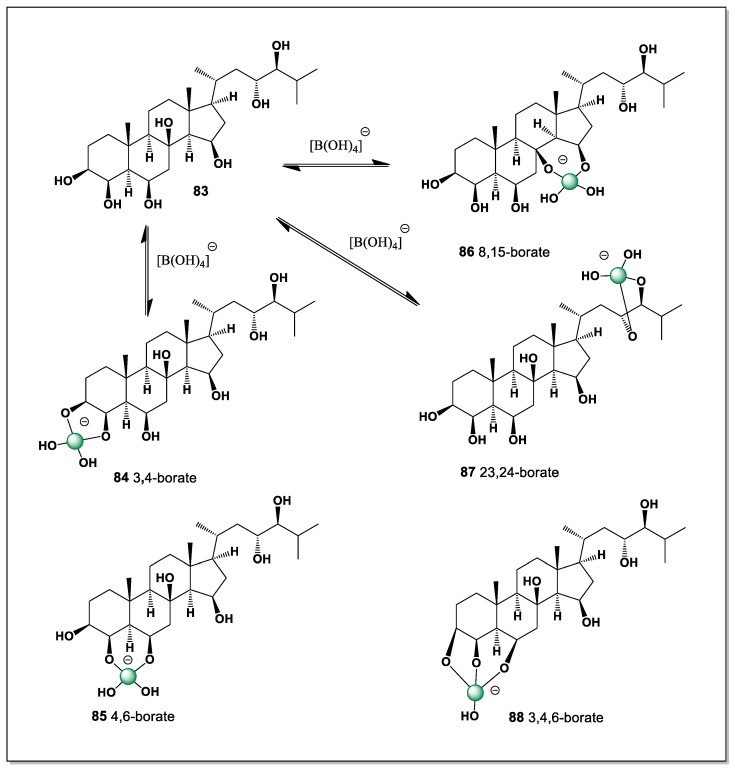
The starfish *Henricia leviuscula* from the Sea of Okhotsk (Russia) contains a polyhydroxysteroid (**83**) bearing seven hydroxyl groups at positions 3, 4, 6, 8, 15, 23, and 24 and forming five boron complex isomers, including two 1,2-diol isomers (**84** and **87**), two 1,3-diol isomers (**85** and **86**), and one mixed 3,4,6-diol isomer (**88**). The coexistence of both 1,2- and 1,3-diol motifs enables diverse boron coordination patterns within a single molecular framework. Such structural diversity highlights the flexibility of boron binding in highly oxygenated starfish steroids. This example further demonstrates how hydroxyl group positioning critically governs the number and type of boron complexes formed.

It inhabits the lower intertidal zone and at depths of 400–600 m. Unlike many predatory sea stars, the blood star feeds primarily on sponges, bryozoans, and small food particles (bacteria) that adhere to the mucus on its body [128,129]. The alcoholic extract of the starfish *Henricia leviuscula*, collected from the Sea of Okhotsk, yielded polyhydroxysteroid (**83**, Figure 20) [129].

Hemolytic activity assays against mouse erythrocytes demonstrated that the compound exhibited membrane-disrupting effects, with HC_50_ values of 2.1 nM. The isolated steroid contains seven hydroxyl groups at positions 3, 4, 6, 8, 15, 23, and 24. The steroid can have at least five isomers, including two 1,2-diol isomers (**84** and **87**), two 1,3-diol isomers (**85** and **86**), and one mixed isomer, 3,4,6 (**88**). The 1,2-diol configuration may be dominant (Figure 20).

The exceptionally high degree of hydroxylation in this polyhydroxysteroid provides multiple potential donor sets for boron coordination, greatly increasing the number of feasible complex isomers. In particular, vicinal 1,2-diol motifs are well known to form the most stable cyclic borate esters, which may explain the predicted dominance of the 1,2-diol–based boron complexes (**84** and **87**). The coexistence of 1,3-diol (**85** and **86**) and mixed chelation modes reflects the broad conformational landscape accessible to such highly oxygenated steroids, despite the intrinsic rigidity of the steroid nucleus. The deep-sea habitat of *Henricia leviuscula*, characterized by elevated pressure and prolonged contact with sediment-associated boron species, may further favor in situ formation of boron–steroid complexes. Together, these features suggest that extreme polyhydroxylation in deep-sea echinoderm steroids enhances both biological activity and coordination versatility, reinforcing their relevance as candidates for marine boronosteroids.

#### 5.2.8. The Sea Urchin *Diadema savignyi*

*Diadema savignyi*, commonly known as the long-spined Savigny’s sea urchin or blue-striped urchin, is a species of marine invertebrate native to tropical waters of the Indo-Pacific and the Red Sea. It inhabits coral reefs, rocky shores, and sandy bottoms at depths of up to 70 m. It feeds primarily on algae, scraping them from corals and rocks, making it an important player in maintaining healthy reef ecosystems [130,131].

**Figure 21:**
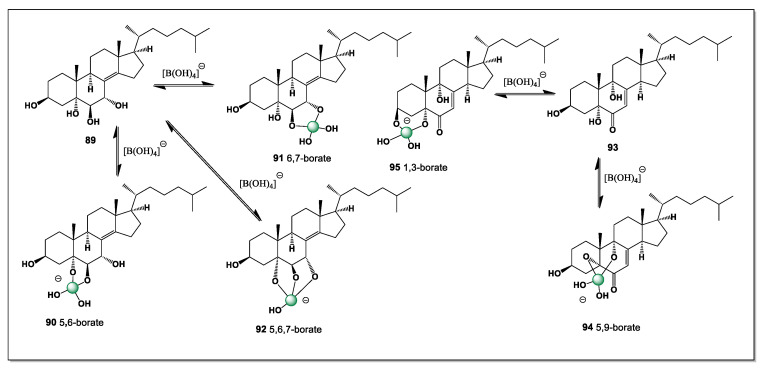
The sea urchin *Diadema savignyi* from tropical waters contains two steroids bearing four hydroxyl groups at positions 3, 5, 6, and 7 that can form three boron complex isomers (**90**–**92**). A second steroid (**93**), with three hydroxyl groups at positions 3, 5, and 9, forms only two boron complex isomers (**94** and **95**). The reduced number of boron complexes in the latter compound reflects fewer available diol coordination motifs. This comparison illustrates how both the number and relative positioning of hydroxyl groups influence boron complex diversity in echinoderm-derived steroids.

Two steroids, Cholest-8(14)-ene3β,5α,6β,7α-tetraol (**89**), which demonstrated cytotoxic activity against prostate cancer cells PC-3 in MTT assay IC_50_ 5.49 µM, and cholest-7-ene-6-one3β,5α,9α-triol (**93**), which had slightly less cytotoxic activity against prostate cancer cells PC-3 in MTT assay IC_50_ 24.4 µM, were discovered and isolated from the sea urchin *Diadema savignyi*. Steroid (**89**), which has four hydroxyl groups at positions 3, 5, 6, and 7, can have three isomers (**90**–**92**, Figure 21), with isomers **90** and **91** predominating. Steroid (**66**), which has three hydroxyl groups at positions 3, 5, and 9, can have only two isomers (**94** and **95**) of boron complexes in combination with 1,3-diols (1 and 3) and (5 and 9), respectively [132].

The presence of 1,3-diol motifs is particularly significant for boron coordination, as these functional groups readily form stable five-membered cyclic borate esters. In steroid (**93**), the 3,5- and 5,9-hydroxyl arrangements generate a well-defined 1,3-diol system that strongly preorganizes the molecule for a single, energetically favored boron-binding mode. This contrasts with vicinal (1,2-) diols, which often allow multiple chelation geometries, highlighting how 1,3-diols impose stricter stereochemical control over boron complex formation in marine steroids.

### 5.3. Soft Corals and Polyhydroxy Steroids

Soft corals (alcyonarians) are marine invertebrates belonging to the order *Alcyonacea*. In contrast to stony corals, they do not possess a massive calcareous skeleton. Instead, structural support is provided by microscopic calcareous spicules, known as sclerites, which impart both flexibility and mechanical stability to the colony. Soft corals are widely distributed in all oceans, occurring from shallow coastal environments to deep-sea habitats. In tropical regions, they frequently dominate reef ecosystems where environmental conditions—such as strong currents, sedimentation, or reduced light availability—are unfavorable for reef-building corals [133,134,135].

Ecologically, soft corals play a critical role by creating complex three-dimensional habitats that provide shelter and refuge for fish and other marine organisms. Although soft corals are generally considered more tolerant to ocean warming than scleractinian corals, they remain vulnerable to ocean acidification, chemical pollution, and other anthropogenic stressors [136,137,138].

From a biomedical perspective, soft corals are of considerable interest as prolific sources of structurally diverse secondary metabolites, including compounds with anti-inflammatory, cytotoxic, and antimicrobial activities. Notably, many coral species accumulate significant amounts of boron, a phenomenon that has been linked to the abundance of hydroxyl-rich natural products within their tissues. The high density of hydroxyl groups is thought to facilitate the formation of stable boron complexes, suggesting a potential biochemical basis for the occurrence of marine boronosteroids and related boron-containing metabolites [2,139,140].

#### 5.3.1. Soft Coral *Nephthea chabrolii*

*Nephthea chabroli*, also known as arborescent coral or cauliflower coral, is a soft, semi-aggressive coral capable of secreting toxins (allelopathic chemicals) to suppress the growth of neighboring corals, especially sensitive SPS corals. The coral primarily obtains nutrition from the photosynthesis of its symbiotic algae. However, it also benefits from supplemental feeding of phytoplankton, microplankton, or other small filter-feeding invertebrates, which should be regularly sprayed over the polyps [141,142,143].

**Figure 22:**
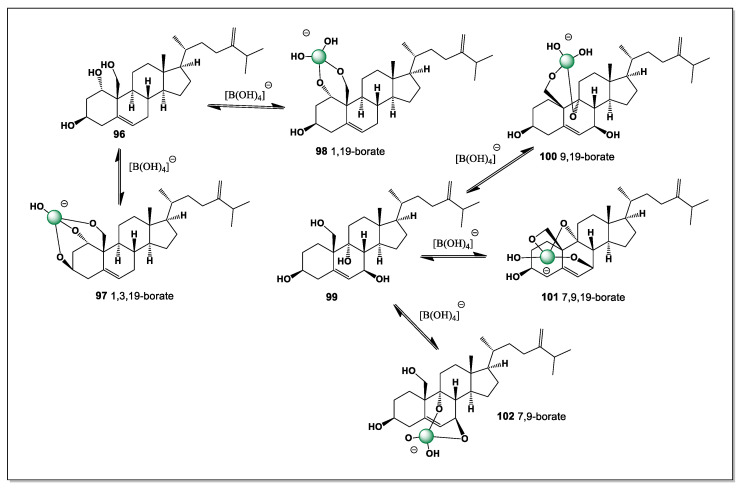
The soft coral *Nephthea chabroli* produces two steroids. The first steroid (**96**) contains three hydroxyl groups at positions 1, 3, and 19 and is capable of forming only two boron complex isomers (**97** and **98**). The second steroid (**99**) bears four hydroxyl groups at positions 3, 7, 9, and 19. The additional hydroxyl group in steroid (**99**) increases the number of potential boron coordination sites. This structural difference is expected to enhance boron-binding versatility compared to steroid (**96**). These findings highlight how incremental changes in hydroxylation patterns modulate boron complex formation in soft coral-derived steroids.

In a study of its extracts, two 19-oxygenated polyhydroxy steroids, 24-methylene cholest-5-en-1α,3β,19-triol (**96**) and 24-methylene cholest-5-en-3β,7β,9α,19-tetrol (**99**) were isolated from the soft coral *Nephthea chabroli* (Figure 22). The first steroid (**96**) contained only three hydroxyl groups at positions 1, 3, and 19, and was capable of forming only two isomers **97** and **98**. The second steroid (**99**) contained four hydroxyl groups at positions 3, 7, 9, and 19, and was capable of having three isomers of boron complexes **100**, **101**, and **102**, with the **98** isomer dominating [144].

The occurrence of 19-oxygenated polyhydroxysteroids in *Nephthea chabroli* highlights the distinctive oxidative biosynthetic pathways operating in soft corals and their role in chemical defense. Although the first steroid possesses a limited number of hydroxyl groups, their favorable spatial arrangement allows selective boron coordination, resulting in only two accessible complex isomers. In the second steroid, the additional hydroxyl functionality increases the number of possible chelation modes; however, conformational preorganization of the steroid nucleus strongly favors formation of a single dominant boron complex. These observations suggest that, even in relatively lightly hydroxylated soft-coral steroids, stereochemical constraints rather than hydroxyl count alone govern the speciation and stability of boron-containing complexes in marine environments.

**Figure 23:**
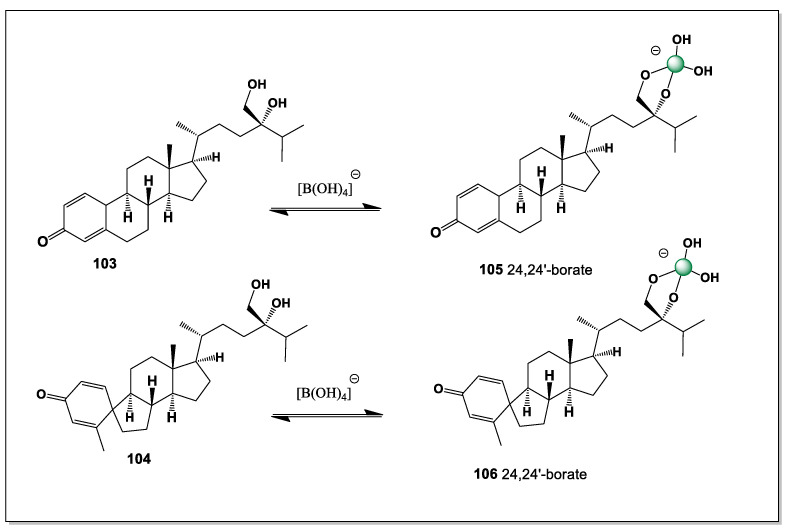
Two steroids with identical side chains were isolated from the soft coral of the genus *Nephthea*. Both steroids possess the same side chain bearing two hydroxyl groups at positions 24 and 24′ and are capable of forming only a single boron complex isomer (**105** and **106**). The identical boron complexation behavior reflects the conserved diol functionality within the side chain. This observation further emphasizes the dominant role of side-chain hydroxyl group arrangement in governing boron coordination in soft coral-derived steroids.

Two other species of the soft coral *Nephthea* sp. produce bioactive steroids. Cytotoxic steroid (**103**) was obtained from the acetone and MeOH extract of the soft coral *Nephthea erecta* [145]. A steroid with a spiro-ring A, B system, named chabrolosteroid C (**104**), was isolated from an organic extract of a Taiwanese soft coral *Nephthea chabrolii* [146]. Both of these steroids contain the same side chain with two hydroxyl groups at positions 24 and 24*, and are capable of only one isomer of the boron complex (**105** and **106**) (Figure 23).

The identical dihydroxylated side chain in both steroids imposes strict stereochemical control over boron coordination, confining complex formation to a single, well-defined isomer. The 24,24′-diol motif provides an optimally spaced donor pair that readily satisfies boron’s preference for cyclic O–B–O chelation. Structural variations in the steroid nucleus, including the unusual spiro A/B ring system in chabrolosteroid C, do not significantly influence boron binding when the coordinating functionality is localized in the side chain. These examples further demonstrate that, in soft-coral steroids, side-chain diols can dominate boron complexation behavior regardless of substantial differences in core steroid architecture.

#### 5.3.2. Soft Octocorals of the Genus *Sinularia*

*Sinularia* is a genus of soft octocorals widespread in the Indo-Pacific region. *Sinularia* periodically shed their skin (a thin layer of mucus) to cleanse themselves of sediment and algae. During this period, the coral may shrink and appear lifeless for several days. Corals in this genus have a fleshy, leathery texture and can assume a wide variety of forms, from massive bushes with finger-like appendages to flat, cabbage-like leaves [147,148,149].

**Figure 24:**
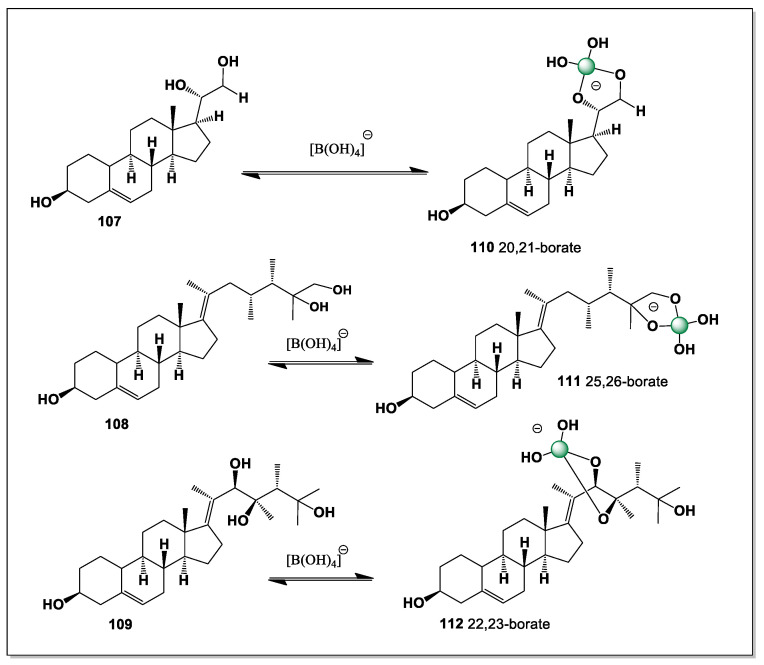
Three steroids were detected in the soft corals *S. mayi*, *S. gibberosa*, *S. dissecta*, and *Sinularia* sp., and it was determined that they each form only a single boron complex isomer. The limited boron isomerism suggests a conserved hydroxyl group arrangement across these steroids. Such structural similarity likely favors a single, energetically preferred mode of boron coordination.

Kobayashi and colleagues studied the soft corals *S. mayi*, *S. gibberosa*, *S. dissecta*, and *Sinularia* sp., collected off the coast of Japan, and identified a number of steroids, including **107**, **108** and **109**, which contain hydroxyl groups in the side chain [150]. So, steroid **108** contains hydroxyl groups at positions 25 and 26, and the second steroid **109** contains hydroxyl groups at positions 22 and 23. Therefore, they can only have one boron complex each (**110**–**112**) (Figure 24).

The presence of vicinal hydroxyl groups confined to the side chain strongly restricts the boron coordination possibilities of these steroids. In both cases, the 25,26-diol in steroid **108** and the 22,23-diol in steroid **109** provide a single, well-defined chelation site that favors formation of only one stable boron complex. The flexible nature of the steroid side chain facilitates optimal alignment of these diols with boron’s preferred coordination geometry, despite the rigidity of the steroid nucleus. These findings from *Sinularia* species reinforce the concept that side-chain diols act as efficient and selective boron-binding motifs in soft-coral steroids.

#### 5.3.3. The Gorgonian Coral *Muricea* cf. *austera*

**Figure 25:**
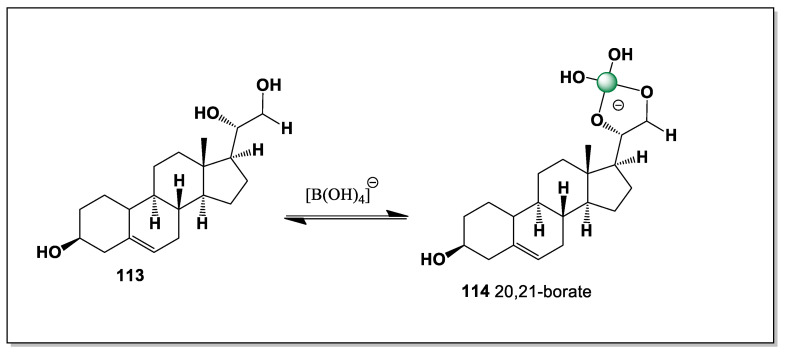
The gorgonian coral *Muricea* cf. *austera* is a source of pregna-5-ene-3,20,21-triol (**113**), which is capable of forming only one boron complex isomer (**114**, Figure 23) through coordination at the side-chain hydroxyl groups at positions 20 and 21. The exclusive formation of a single boron complex indicates a well-defined 1,2-diol arrangement in the side chain. This structural specificity likely promotes selective and stable boron coordination.

*Muricea* cf. *austera* is a species of gorgonian coral (sea fans) in the family Plexauridae, found primarily in shallow waters of the eastern Pacific Ocean, including the Gulf of California [151,152]. This species is known for its role as a source of novel bioactive compounds. This species is native to the eastern Pacific Ocean, and the genus *Muricea* as a whole is characterized by high species diversity in this region. The trihydroxy (3, 20, and 21) sterol, pregna-5-ene-3,20,21-triol (**113**), has been isolated from the Gulf of California gorgonian *Muricea* cf. *austera*. This steroid is capable of forming only one isomer of boron complex (**114**, Figure 25) in the side chain (20 and 21) [153].

The restriction to a single boron complex isomer reflects the localization of hydroxyl functionality within the steroid side chain rather than on the rigid tetracyclic nucleus. In pregna-5-ene-3,20,21-triol, the 20,21-diol provides the only suitably oriented donor pair for stable boron chelation, while the 3β-hydroxyl group remains conformationally isolated. This side-chain–focused coordination mode is consistent with the relatively flexible geometry of C20–C21, which readily accommodates boron’s preferred coordination environment. The example from *Muricea* cf. *austera* further illustrates how modestly hydroxylated gorgonian sterols can still participate in selective boron complex formation when functional groups are optimally positioned.

### 5.4. Marine Fungal Endophytes

Marine fungal endophytes are fungi that inhabit the internal tissues of marine organisms—including algae, mangroves, sponges, and corals—without causing disease. They represent a prolific source of structurally novel and biologically active secondary metabolites, such as polyketides, alkaloids, and terpenoids, many of which exhibit potent anticancer, antimicrobial, antiviral, and antioxidant activities. These microorganisms play important roles in maintaining host homeostasis and adaptation to environmental stressors, while also offering significant promise for pharmaceutical, industrial, and agricultural applications [154,155,156,157]. The unique and often extreme marine environments in which these endophytes thrive drive the evolution of uncommon biosynthetic pathways, resulting in chemical scaffolds rarely found in terrestrial fungi. Advances in genomics, metabolomics, and cultivation techniques have greatly enhanced the discovery and characterization of these metabolites. Moreover, marine fungal endophytes are increasingly recognized as sustainable resources for natural product drug discovery. Continued exploration of their diversity and symbiotic interactions is expected to yield new bioactive compounds with translational biomedical potential [157,158,159].

These fungi are essential components of marine biodiversity, coevolving with their hosts and likely contributing to their homeostasis and defense mechanisms. While over 1100 species are documented, potentially 10,000 exist. They are frequently found in mangroves (second largest group) and as algicolous (algae-associated) endophytes. They are considered “biosynthetic engines,” producing unique chemical structures with antiviral, neuroprotective, and immunosuppressant properties. Beyond medicine, these fungi are studied for bioremediation (e.g., plastic degradation) and in vitro biosynthesis of nanoparticles [160,161,162].

**Figure 26:**
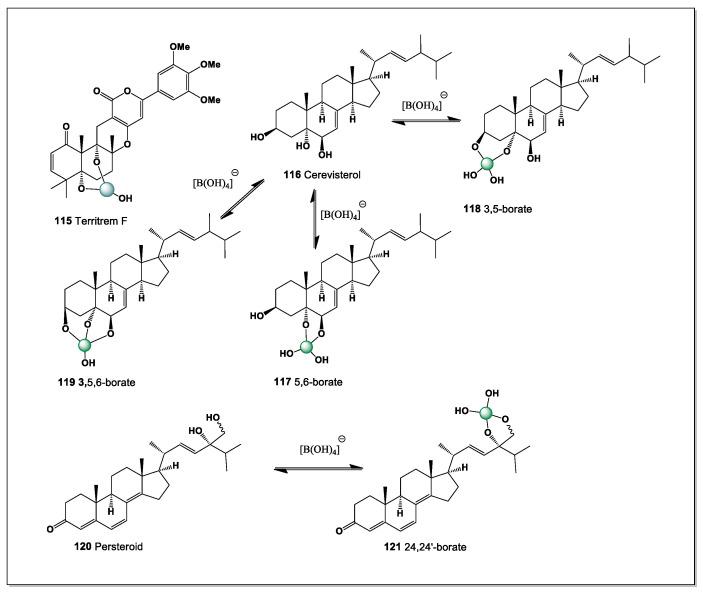
Three different fungal metabolites are shown. Territrem F (**115**) is a borate 1,3-diol meroterpenoid containing a unique borate ring system and represents a rare example of a naturally occurring boron-containing secondary metabolite, isolated from a soft coral-associated fungus (*Alternaria* sp.). Another steroid, cerevisterol, is produced by the marine fungus *Penicillium levitum*, while a third steroid, persteroid, is produced by the marine-derived fungus *Penicillium* sp. ZYX-Z-143. Together, these compounds illustrate the remarkable chemical diversity of marine and marine-associated fungi as sources of both boron-containing and boron-complexing metabolites. They also highlight distinct biosynthetic strategies through which fungal steroids and meroterpenoids can engage in boron coordination. Such findings reinforce the importance of fungi in expanding the known chemical space of natural boron-containing bioactive molecules.

Driman borate, territrem F (**115**, see Figure 26), and its diol precursor, territrem B, were isolated from a soft coral-associated fungus (*Alternaria* sp.) [163]. This compound possesses a unique borate ring system and demonstrates significant inhibitory activity against synchronous Ca^2+^ oscillations and epileptic discharges induced by 4-aminopyridine. Territrem F borate (**115**) is one of the few boron complexes isolated from natural sources. Broadly, this finding highlights marine-derived fungi as rare yet valuable producers of naturally occurring boron-containing metabolites. The incorporation of boron into the drimane framework appears to confer distinctive neuroactive properties, suggesting a functional role for boron beyond structural modification. These observations support the idea that boron complexation can enhance or modulate biological activity, reinforcing its relevance in natural product–based drug discovery.

Territrem F is a borate 1,3-diol meroterpenoid containing a unique borate ring system and represents a rare example of a naturally occurring boron-containing secondary metabolite. It belongs to the territrem family of fungal meroterpenoids and is a metabolite secreted by fungi of the genus *Alternaria*, specifically the coral-associated strain ZH-15. Territrem F (Figure 25) is a structural derivative of the better-known territrem B, a potent and irreversible acetylcholinesterase inhibitor with pronounced tremorgenic activity, suggesting that boron incorporation may significantly alter both the mechanism and profile of biological activity within this compound class [164].

A steroid called cerevisterol (22E)-ergosta-7,22-diene-3β,5α,6β-triol, (**116**) produced by the marine fungus *Penicillium levitum*, exhibits cytotoxic activity against hepatocellular carcinoma (HepG2) and lung carcinoma (A549) cells [165]. The compound contains three hydroxyl groups at positions 3, 5, and 6 and is capable of forming three boron complex isomers (**117**–**119**), with the dominant species corresponding to the 1,2-diol at positions 5 and 6 (**118**). The hydroxyl groups at positions 3 and 5 constitute a 1,3-diol motif, which in this case gives rise to minor boron complex isomers. The preference for 1,2-diol boron coordination suggests a more favorable geometric and electronic environment compared to the 1,3-diol arrangement. Such selectivity may influence the stability and abundance of the resulting boron complexes. Boron complexation could also modulate the cytotoxic properties of cerevisterol by altering molecular conformation and target interactions. These findings further illustrate the capacity of marine fungal steroids to generate multiple boron-containing derivatives with potentially distinct biological activities.

Cerevisterol is a bioactive ergostanoid widely distributed among fungi, including *Saccharomyces cerevisiae*, *Agaricus blazei*, and species of *Xylaria*, and was originally identified in the 1930s [166]. It exhibits a broad spectrum of biological activities, including antimicrobial, anti-inflammatory, and anticancer effects, such as inhibition of proliferation in multiple cancer cell lines. The anti-inflammatory activity of cerevisterol is associated with suppression of lipopolysaccharide-induced nitric oxide production and downregulation of proinflammatory cytokines such as TNF-α and IL-6. Its cytotoxicity against cancer cells (e.g., MCF-7 and Caco-2) and activity against bacterial and fungal pathogens underscore its pharmacological versatility [166,167,168]. The occurrence of cerevisterol in marine-derived fungi suggests ecological conservation of its biosynthetic pathway across terrestrial and marine environments. Notably, boron complexation appears to enhance its biological potency, indicating that cerevisterol boron complexes may represent more active pharmacological entities than the parent non-borate form.

A steroid named persteroid (**120**) was isolated from the marine fungus *Penicillium* sp. ZYX-Z-143 and exhibited inhibitory activity against nitric oxide production in LPS-stimulated RAW 264.7 macrophages, with an IC_50_ value of 25.81 µM [169]. The compound contains two hydroxyl groups at positions 24 and 24′, forming a 1,2-diol motif and, accordingly, generates only a single boron complex isomer (**121**). The exclusive formation of one boron complex reflects the well-defined diol geometry within the side chain. Such structural simplicity may favor stable boron coordination with minimal conformational variability. This example further supports the relationship between diol arrangement and predictable boron complexation behavior in marine fungal steroids.

C25 steroids bearing a bicyclo[4.4.1] A/B ring system constitutes a rare and structurally distinctive family currently comprising sixteen known members. These compounds differ primarily in the structure of their C-17 side chains and are grouped into four subtypes: cyclocitrinol, isocyclocitrinol, neocyclocitrinol, and precyclocitrinol. The first representative, cyclocitrinol, was originally isolated from the terrestrial fungus *Penicillium citrinum* and described as a novel sisterpene; however, subsequent re-isolation from a sponge-derived strain of *P. citrinum* led to a structural revision identifying it as a bicyclo[4.4.1] A/B ring steroid with a trans-22,23 double bond [170,171].

**Figure 27:**
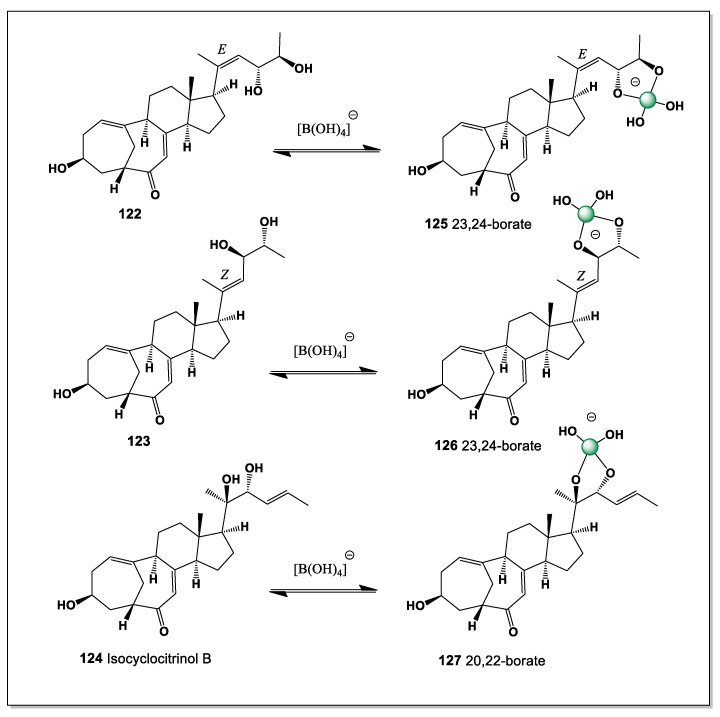
Unique C25 steroids (**122**–**124**) featuring an unusual bicyclo[4.4.1] A/B ring system were identified in extracts of the marine strain *Penicillium purpurogenum* G59. All of these steroids contain three hydroxyl groups yet form only a single boron complex isomer in the side chain. The exclusive formation of one isomer reflects a highly constrained and stereochemically defined side-chain architecture. This structural rigidity likely promotes selective boron coordination and contributes to the distinct chemical behavior of these marine fungal steroids.

The cyclocitrinol subtype now includes several analogs, such as cyclocitrinol, 12-hydroxycyclocitrinol, 20-O-methylcyclocitrinol, 24-epi-cyclocitrinol, 20-O-methyl-24-epi-cyclocitrinol, and 24-oxiocyclocitrinol (Figure 27). Members of the isocyclocitrinol subtype are characterized by a trans-23,24 double bond and include isocyclocitrinols A and B as well as 22-acetylisocyclocitrinol.

Neocyclocitrinol was initially obtained as an unresolved mixture of 23,24-epimers with an undetermined 20,22 double-bond geometry; later studies resolved all four diastereomers—neocyclocitrinols A–D—and established their absolute configurations, including E-configured 20,22 double bonds, along with two methylated derivatives, erythro- and threo-23-O-methylneocyclocitrinols. In contrast, the precyclocitrinol subtype is represented by a single compound, precyclocitrinol B, distinguished by a unique 20,22-epoxy moiety [170,171,172,173,174]. These findings demonstrate that marine-derived species of the genus *Penicillium* are capable of synthesizing structurally diverse and biologically active cyclocitrinol-type steroids, underscoring their importance as a source of novel marine natural products.

The marine strain Penicillium purpurogenum G59 is a source of 25-steroids (**122**–**124**) featuring an unusual bicyclo[4.4.1] A/B ring system and a Z-configuration of the C20–C22 double bond (**127**) (Figure 27). Production of these metabolites in the AD-1-2 mutant extract was achieved by activating silent biosynthetic pathways in the original G59 strain through DES mutagenesis [170].

All of the steroids form a single boron complex isomer (**125**–**127**) via side-chain coordination at C20 and C22, in conjunction with hydroxyl groups at C23 and C24, representing *cis*-1,2-diol borate complexes (Figure 25). The exclusive formation of one boron isomer indicates a highly defined and rigid side-chain geometry. Such stereochemical constraint favors selective boron coordination and limits alternative binding modes. These compounds further demonstrate how genetically induced metabolic activation can uncover structurally unique steroids with predictable boron complexation behavior. They also expand the repertoire of fungal-derived *cis*-1,2-diol systems capable of stable boron incorporation.

**Figure 28:**
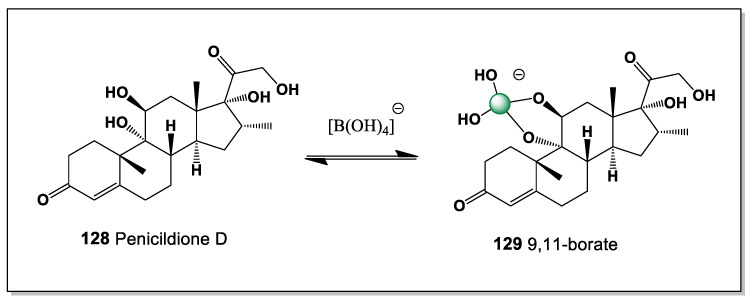
The soft coral-derived fungus *Penicillium* sp. SCSIO41201 produces the steroid penicildione D (**128**), which contains four hydroxyl groups but forms only a single boron complex isomer through coordination at positions 9 and 11 (**129**). This restricted boron complexation indicates a rigid spatial arrangement of the cis- 9,11-diol motif. Such structural constraint may play a role in stabilizing the molecule and influencing its pronounced cytotoxic activity.

Penicildione D (**128**, Figure 28), a rare steroid, was isolated from the soft coral-derived fungus *Penicillium* sp. SCSIO41201 cultured in a 1% NaCl PDB medium. It exhibited pronounced cytotoxic activity against HL-60, K562, MOLT-4, ACHN, 786-O, and OS-RC-2 cell lines, with IC_50_ values of 5.23, 4.12, 4.31, 23.55, 7.65, and 10.81 μM/L, respectively. The compound contains four hydroxyl groups but forms only a single boron complex isomer via coordination at positions 9 and 11 (**129**) [175]. The restriction to one boron isomer suggests a highly specific *cis*-1,2-diol arrangement at C9 and C11. Such regioselective boron complexation may contribute to the stability of the molecule. The strong cytotoxic profile highlights penicildione D as a promising lead for anticancer research. This example further underscores marine-derived *Penicillium* species as valuable sources of structurally constrained, bioactive steroidal scaffolds.

Species of *Aspergillus* represent a major and ecologically important component of marine fungal communities and are commonly found in habitats such as mangrove humus, coral reef symbioses, and deep-sea sediments. To survive in these environments, marine *Aspergillus* species have developed sophisticated adaptive strategies to cope with extreme conditions, including elevated salinity, high hydrostatic pressure, and reduced oxygen availability. These environmental pressures have driven the evolution of secondary metabolic pathways that differ markedly from those of terrestrial counterparts, facilitated by horizontal gene transfer and extensive reorganization of metabolic networks, ultimately resulting in the biosynthesis of structurally unique natural products. Surveys of marine microbial metabolites indicate that *Aspergillus* species are the most intensively investigated marine fungi, accounting for approximately 31% of newly reported natural products from marine fungal sources [176,177,178].

Marine-derived *Aspergillus* strains are particularly prolific producers of chemically diverse and biologically active secondary metabolites, many of which show promising pharmacological properties. Marine sediments represent the predominant source of these strains (about 25.9%), followed by isolates obtained from sponges and corals. The wide range of chemical scaffolds produced by marine *Aspergillus* has significantly expanded the pool of candidates for drug lead discovery. Many of these metabolites display antimicrobial, anticancer, antiviral, and anti-inflammatory activities. In addition to biomedical applications, some compounds exhibit potent activity against agricultural pathogens. Collectively, these findings establish marine *Aspergillus* fungi as a valuable and sustainable resource for future drug and agrochemical development [177,178,179].

**Figure 29:**
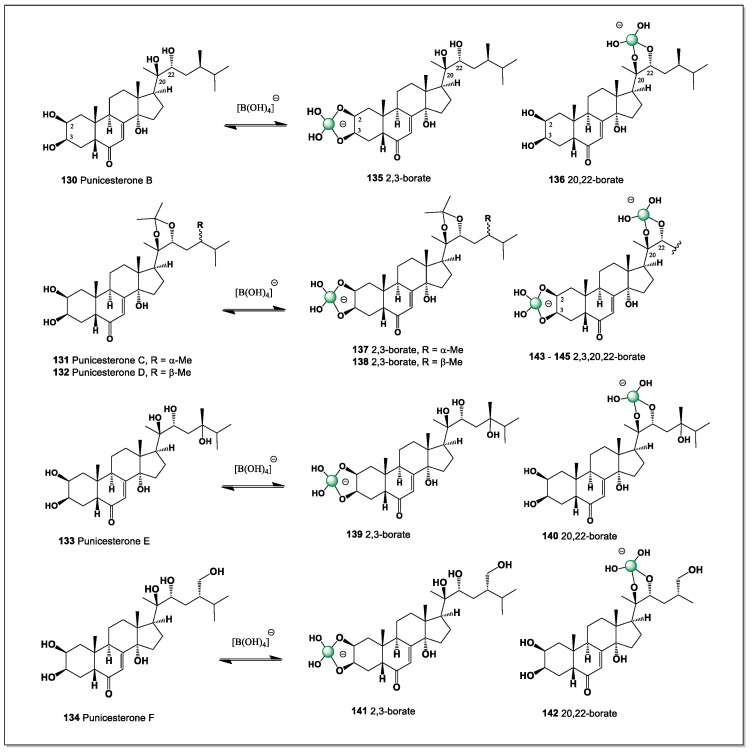
The deep-sea-derived fungal strain *Aspergillus puniceus* SCSIO z021 produces several bioactive steroids (**130**–**134**) capable of forming one to three boron complex isomers (**135**–**145**). The variation in boron isomer number reflects differences in hydroxyl group distribution and diol accessibility among these steroids. These compounds further demonstrate how deep-sea fungi generate structurally adaptable metabolites whose boron coordination behavior is finely tuned by molecular architecture.

The deep-sea-derived fungal strain *Aspergillus puniceus* SCSIO z021 is a source of steroids called punicesterones B–G (**130**–**134**), which showed cytotoxicity and could reduce intracellular lipid accumulation (Figure 29). 

Additionally, antibacterial activity assays indicated that compounds **130** and **131** exhibited moderate antibacterial activity against five bacterial strains [180].

Punicesterone B (**130**) contains five hydroxyl groups at positions 2, 3, 14, 20, and 22 and typically forms two boron complex isomers (**135** and **136**), although an additional isomer (**143**) may arise under conditions of elevated boron availability in the fungal cells or surrounding microenvironment.

In contrast, punicesterones C (**131**) and D (**132**), in which the hydroxyl groups at positions 20 and 22 are blocked, each form only a single boron complex isomer (**137** and **138**). Punicesterones E (**133**) and F (**134**) generate three boron complex isomers each—**139**, **140**, and **144** for E, and **141**, **142**, and **145** for F. These patterns illustrate how both the number and accessibility of diol motifs directly control boron complex diversity. Blocking key hydroxyl groups effectively restricts boron coordination and reduces isomer formation. Conversely, increased hydroxylation and flexible diol arrangements favor multiple coordination modes. Such sensitivity to local boron concentration further supports the concept of dynamic, environment-dependent boron–steroid speciation.

## 6. Biological Activity of Steroid–Borate Complexes

Steroid–borate complexes represent a relatively unexplored class of bioactive molecules whose biological properties may differ substantially from those of the parent steroids. Formation of borate esters with vicinal diols or polyhydroxylated regions of steroid molecules can alter several key physicochemical parameters, including polarity, hydrogen-bonding capacity, conformational rigidity, and membrane affinity. These changes may influence the interaction of steroid frameworks with biological targets such as membrane receptors, enzymes, and ion channels. In marine organisms, many polyhydroxylated steroids—particularly those isolated from sponges, echinoderms, and corals—exhibit a wide spectrum of biological activities, including cytotoxic, antimicrobial, antiviral, anti-inflammatory, antifouling, and antiparasitic effects [49,80,81]. When borate coordination occurs, these activities may be modified through changes in molecular recognition or cellular uptake. For example, borate ester formation can stabilize certain conformations of polyhydroxy steroids, potentially enhancing binding to protein targets or modulating membrane permeability. In addition, boron-containing natural products and borate complexes are known to interact with biological systems through Lewis acid–base interactions with nucleophilic residues, suggesting that steroid–borate complexes may display unique modes of action not observed for the parent steroids. Although most reported biological effects of marine polyhydroxysteroids are currently described qualitatively, available data indicate a wide range of potencies, from weak to strong activities depending on structural features such as the number and position of hydroxyl groups, presence of sulfate substituents, side-chain oxidation patterns, and overall stereochemistry. Systematic evaluation of these compounds, including determination of quantitative parameters such as IC_50_, MIC, or other dose–response values, will be essential for understanding the precise structure–activity relationships governing steroid–borate complexes. Future studies should therefore integrate chemical characterization of borate-binding motifs with biological assays to determine whether boron coordination enhances, attenuates, or redirects the biological activity of marine steroids. Such work may ultimately clarify whether boron complexation represents merely a chemical modification of existing steroid scaffolds or a distinct biochemical strategy influencing ecological interactions and cellular signaling in marine organisms [49,80,81].

### 6.1. Comparison of Steroid–Borate Complexes with Boron-Containing Antibiotics

A useful starting point is that *steroid–borate complexes* and the classic boron-containing polyketide antibiotics belong to the same broad chemical universe—both rely on boron–oxygen coordination—but they differ profoundly in origin, architecture, stability, and biological function. Steroid–borate complexes are usually conceived as reversible adducts formed when a polyhydroxylated steroid presents a suitable diol or polyol array to boric acid/borate in solution. By contrast, compounds such as boromycin, borophycin, aplasmomycin, tartrolon B, and hyaboron are discrete natural products in which boron is already built into the isolated metabolite, usually as part of a macrodiolide or related oxygen-rich framework that has evolved to accommodate boron as a structural element [80,81,180,181,182,183,184,185].

One factual correction is important before making the comparison: not all of these compounds are produced by marine bacteria. Aplasmomycin is clearly marine-derived, having been isolated from *Streptomyces griseus* from sea sediment in Sagami Bay [186,187,188]. Borophycin was reported from a marine strain of the cyanobacterium *Nostoc linckia*, so it is marine and microbial, but not a heterotrophic bacterium in the usual actinomycete sense [189,190]. Boromycin is classically associated with *Streptomyces antibioticus* from soil, not a marine source [80,81]. Tartrolon B was first reported from the myxobacterium *Sorangium cellulosum* and later borated tartrolons were also found from a shipworm symbiont, giving the family both terrestrial and marine-associated examples [81,191]. Hyaboron was isolated from the myxobacterium *Hyalangium minutum* and is not generally described as a marine metabolite [192,193]. So the cleaner statement is that these are a remarkable family of naturally occurring boron-containing polyketides, some of which are marine or marine-associated [81].

The biggest chemical contrast is how boron is incorporated. In proposed steroid–borate complexes, boron is expected to bind to pre-existing vicinal diols, 1,3-diols, or clustered hydroxyl arrays on a steroid scaffold, often in a dynamic and reversible equilibrium controlled by pH, boron concentration, solvent, and local microenvironment. In boromycin-like antibiotics, boron is not an incidental guest but part of a preorganized natural pharmacophore. These compounds are often described as Boeseken-type borate complexes or closely related borate-centered architectures in which boron locks an oxygen-rich polyketide into a specific three-dimensional arrangement. In other words, steroid–borate complexes are best viewed as conditionally formed coordination species, whereas boromycin, aplasmomycin, borophycin, tartrolon B, and hyaboron are best viewed as native boron-dependent natural products.

A second major difference is the role of the organic scaffold. Polyhydroxylated marine steroids are typically compact, rigid, tetracyclic frameworks in which hydroxyl groups are displayed on one or more faces of the steroid nucleus. That rigidity can favor borate binding, but the boron-binding motifs are usually localized patches on a larger hydrophobic scaffold. In the boron-containing polyketide antibiotics, the ligand environment is much more oxygen-rich, macrocyclic, and polydentate. Macrodiolide frameworks provide a cavity or binding environment in which boron is coordinated by multiple oxygen donors in a way that is much more integral to the global conformation of the molecule. Thus, in steroids, boron often acts conceptually as a modifier of an existing scaffold; in polyketide boron antibiotics, boron is closer to a central organizing atom.

This structural difference leads directly to a difference in stability and speciation. Steroid–borate complexes in seawater are likely to exist as equilibrating species, and in many cases one should expect a population of free steroid, mono-borate adduct, and possibly alternative borate-bound forms. Their abundance may vary with pH and competing ligands. The naturally occurring boron-polyketides, by contrast, are isolated as defined compounds, which means that the boron-containing form is sufficiently stable to survive biosynthesis, isolation, and purification. Even when desboro analogs exist, as in the aplasmomycin literature, the borated species remains a chemically distinct entity with a recognizable structure. That makes these antibiotics a much stronger demonstration of stable, biologically meaningful boron incorporation than the still largely hypothetical marine steroid–borate equilibria.

The comparison is especially instructive in terms of biological function. For steroid–borate complexes, the most plausible biological consequences are modulation of physicochemical properties: altered solubility, changed membrane partitioning, modified hydrogen-bonding patterns, and possible shifts in receptor or enzyme recognition. The boron might tune the activity of a steroid, but it is unlikely to create an entirely new mechanism unless it strongly changes conformation or target binding. In contrast, the naturally occurring boron-containing polyketide antibiotics are famous because boron appears to be part of the mechanistic core. Boromycin is widely recognized as a potassium ionophore that dissipates ion gradients and membrane potential [81]. Hyaboron and related borated natural products were likewise reported to stimulate NLRP3-dependent IL-1β maturation, with the activity most likely arising from potassium ionophore behavior; hyaboron also showed antibacterial and antiparasitic effects. Tartrolon B has similarly been linked to the boron-binding macrodiolide family and to strong anti-Gram-positive and cytotoxic activities [192].

So, from a pharmacological standpoint, the two families occupy different conceptual niches. Steroid–borate complexes are best considered as boron-modulated derivatives of known bioactive steroids, where boron may fine-tune potency, selectivity, transport, or ecological behavior. Boromycin, aplasmomycin, borophycin, tartrolon B, and hyaboron are better viewed as intrinsically boron-dependent natural products whose boron-centered architecture is part of the reason they function at all [80,81,182]. In medicinal chemistry language, boron is likely to be auxiliary in the first class and constitutive in the second. That distinction is crucial when drawing analogies: one should not assume that because a marine steroid can bind borate, it will behave like a boromycin-like ionophore.

Looking at the named compounds individually makes the comparison sharper. Boromycin was the first well-defined natural organoboron compound and remains the prototype for boron-containing ionophoric antibiotics. It comes from *Streptomyces antibioticus* and is strongly associated with anti-Gram-positive activity and ion transport across membranes. Relative to a steroid–borate complex, boromycin is much more architecturally committed to boron: remove the boron-centered organization and you are no longer dealing with a modestly modified hydrophobic signaling scaffold, but with a substantially altered ionophoric system [80].

Aplasmomycin is perhaps the most relevant comparator if one wants a marine example. It was isolated from a *Streptomyces griseus* strain from sea sediment in Sagami Bay and was reported to inhibit Gram-positive bacteria, including mycobacteria, and to show antiplasmodial activity. Because it is marine-derived and boron-containing, aplasmomycin demonstrates that stable boron-centered antibiotic architectures are compatible with marine microbial metabolism. Yet even here the contrast with steroid–borate complexes remains strong: aplasmomycin is not simply a marine polyol that happened to trap borate from seawater, but a true natural product whose boron-containing form is part of its identity [80,81,186,187,188].

Borophycin extends the comparison into marine cyanobacterial chemistry. It was described as a new Boeseken complex of boric acid from a marine strain of *Nostoc linckia*, and later summaries also note its occurrence in *Nostoc spongiaeforme var. tenue*. It is notable mainly for potent cytotoxicity, rather than for the classic antibacterial ionophore profile associated with boromycin. This shows that the boron-containing polyketide family is not functionally uniform: boron can support not only ionophore-type antibiotics but also strongly cytotoxic macrocycles. Compared with steroid–borate complexes, borophycin illustrates how a boron-containing natural product can have a highly specialized and potent biological profile that is difficult to infer merely from “borate binding” alone [189,190].

Tartrolones are particularly informative because the family includes both boron-free and boron-containing members. The classic study on *Sorangium cellulosum* reported tartrolons A and B, with tartrolon B containing boron, and explicitly noted that its boron-binding region is identical with that of boromycin and aplasmomycin [80,81,192]. Later work on a shipworm symbiont further emphasized borated tartrolons as marine-associated specialized metabolites. This family therefore provides a bridge between the two concepts: it shows a scaffold that can exist in different boronation states, making it a particularly useful model for thinking about what boron does to conformation and activity. In that sense, tartrolones are closer than boromycin to the question posed by steroid–borate complexes, because they show more directly how the same general framework may change on boron incorporation [80,81,182].

Hyaboron adds an important modern example. It was reported as a new asymmetric boron-containing macrodiolide from *Hyalangium minutum* and was shown to activate the NLRP3 inflammasome while also displaying antibacterial and antiparasitic activities; the mechanism was linked to potassium ionophore-like behavior. Compared with steroid–borate complexes, hyaboron again highlights that naturally occurring boron-polyketides often act through membrane and ion-homeostasis mechanisms, not simply through subtle receptor modulation. This makes them biologically more dramatic and mechanistically more disruptive than what one would usually predict for reversible borate binding to a steroid [192,193].

If one asks what these comparisons imply for marine steroid research, the answer is that steroid–borate complexes should not be sold as “steroid versions of boromycin.” That would overstate the analogy. The better formulation is that boromycin, aplasmomycin, borophycin, tartrolon B, and hyaboron prove that boron can be central to natural product architecture and activity, including in marine or marine-associated systems. They therefore provide a compelling precedent that boron binding in marine chemistry is biologically real, not speculative. But the expected consequence for steroids is probably subtler: boron may regulate speciation, conformation, transport, and target engagement, whereas in the polyketide macrodiolides it often underpins a preassembled ionophoric or cytotoxic framework [80,81].

A final comparison concerns ecology. Marine steroid–borate complexes, if they exist widely, may function as context-dependent chemical states shaped by seawater chemistry, local pH, and borate availability; they could subtly alter deterrence, membrane behavior, or signaling in marine invertebrates. The boron-containing polyketide antibiotics, by contrast, are much more likely to act as offensive or defensive specialized metabolites produced by microbes in competition with other organisms. In other words, marine steroid–borate complexes fit a model of environmentally modulated chemical ecology, whereas boromycin-like compounds fit a model of genetically encoded boron-dependent weaponry. That may be the most useful conceptual distinction of all.

### 6.2. Marine Holobionts and Steroid–Borate Complexes

Marine sponges are now widely recognized as holobionts that host dense, taxonomically diverse microbial consortia, sometimes comprising a substantial fraction of sponge biomass. These communities include bacteria, archaea, cyanobacteria, microalgae, and fungi, and many sponge-associated natural products are now thought to be made by symbionts rather than by sponge cells alone [194,195,196,197,198]. That general framework is important here, because it makes sponge tissues plausible sites not only for the biosynthesis of specialized metabolites, but also for secondary chemical transformations such as borate complexation in the seawater-exposed sponge microenvironment.

Within this microbiome, Streptomyces-like actinomycetes are well documented sponge associates. Multiple studies have isolated *Streptomyces* strains from marine sponges and shown that these bacteria produce antibacterial, antiviral, anticancer, antioxidant, and antibiofilm metabolites. Sponge-associated *Streptomyces* are therefore not hypothetical contributors to sponge chemistry; they are experimentally demonstrated members of the sponge metabolite-producing consortium. This matters because boron-containing polyketide antibiotics such as aplasmomycin are produced by *Streptomyces griseus*, and boromycin is classically associated with *Streptomyces antibioticus*. Even though those famous boron-containing antibiotics were not originally isolated from sponges themselves, the sponge microbiome clearly contains related actinomycete lineages capable of rich secondary metabolism, making sponge-associated production of analogous boron-centered metabolites chemically and biosynthetically plausible [199,200,201,202,203].

A second relevant symbiont group is the cyanobacterial lineage represented by *Nostoc* and closely related forms. Sponges are known to host cyanobacterial symbionts, and borophycin has been reported from marine cyanobacterial material including *Nostoc linckia* and *Nostoc spongiaeforme* var. *tenue*. This is especially significant because it provides a direct example in which a boron-containing natural product is associated with a cyanobacterial taxon linked to sponge-related or marine settings. Thus, in a sponge holobiont, cyanobacterial partners are not merely phototrophic residents; they may also be contributors to a boron-reactive chemical network that includes highly oxygenated metabolites and, potentially, boron-containing antibiotics [204,205,206,207,208].

The case for myxobacteria is more nuanced. Marine myxobacteria are real and metabolically rich, and reviews note that some metabolites first found in marine sponges also occur in terrestrial or marine myxobacteria, raising the possibility that certain “sponge natural products” may actually derive from myxobacterial symbionts or close biosynthetic relatives. At the same time, the evidence for stable, routine myxobacterial symbiosis in sponges is less direct than it is for actinomycetes or cyanobacteria. Therefore, the most defensible statement is that myxobacteria are credible biosynthetic analogs and possible contributors, rather than universally proven sponge symbionts in the same sense as well-studied sponge-associated *Streptomyces* or cyanobacteria. This distinction is important for keeping the discussion chemically ambitious but microbiologically accurate [209,210,211,212].

Against this microbiological background, the comparison with boron-containing polyketide antibiotics becomes especially informative. Boromycin, aplasmomycin, borophycin, tartrolone-family borates, and hyaboron all show that natural systems can use boron as a structural component of stable, bioactive metabolites. In these compounds, boron is not an incidental environmental contaminant; it is part of a defined molecular architecture, usually coordinated by oxygen-rich polyketide frameworks. Aplasmomycin is a *Streptomyces* product, borophycin is linked to *Nostoc*, tartrolon-type borated metabolites occur in symbiotic systems such as a shipworm symbiont, and hyaboron comes from a myxobacterium. Collectively, these examples establish a strong precedent that the microbial partners relevant to sponge holobionts—or closely related to them—are chemically capable of making authentic boron-containing antibiotics [80,81,180,181,182,183,184,185].

This precedent supports a broader ecological hypothesis for marine sponges: the sponge body may function as a boron-enabled chemical reactor in which two related but distinct processes occur simultaneously. The first is true biosynthesis of boron-containing antibiotics by microbial symbionts, especially oxygen-rich polyketides whose structures are preorganized to trap boron as a stable component. The second is post-biosynthetic borate complexation of sponge or symbiont metabolites that contain vicinal diols, 1,3-diols, or clustered hydroxyl groups. Because seawater contains abundant dissolved boron, predominantly as borate under mildly alkaline conditions, and because sponge mesohyl provides a crowded microenvironment enriched in polyhydroxylated natural products, both processes are chemically plausible in the same organismal setting [213,214,215].

This is where steroid–borate complexes become especially relevant. Marine sponges are famous for producing polyhydroxylated steroids and secosteroids, many of which contain diol arrays suitable for reversible borate binding. If the same sponge also harbors boron-competent symbionts such as *Streptomyces* or cyanobacterial partners, the holobiont may contain both: (i) Microbially synthesized boron-containing antibiotics, and (ii) Host- or symbiont-derived steroidal borate complexes formed through environmental borate coordination.

These are chemically different phenomena—one biosynthetic, one equilibrium-based—but they are not mutually exclusive. On the contrary, they may coexist in the same sponge and contribute together to chemical defense, microbial competition, signaling, and metabolite trafficking.

A particularly attractive idea is that sponge symbionts may participate in steroid–borate chemistry indirectly as well as directly. Direct participation would mean producing boron-containing metabolites themselves. Indirect participation could include shaping the local pH, redox state, metabolite pool, and concentration of competing polyols, all of which can influence borate speciation and ester formation. Cyanobacterial photosynthesis, microbial respiration, and localized enzymatic release of polyhydroxylated metabolites may all help determine whether a sponge steroid remains in its free form or shifts into a boron-bound state. In this view, the sponge microbiome is not just a source of antibiotics; it is part of the chemical context that governs borate complexation equilibria.

The ecological implications are substantial. Boron-containing antibiotics from symbionts could act as defensive or competitive metabolites against invading bacteria, fouling organisms, or predators, while steroid–borate complexes could modify membrane affinity, conformation, solubility, and receptor interactions of sponge steroids. The result may be a layered defense system in which microbial boron-polyketides provide direct antimicrobial or ionophoric effects, whereas steroid–borate complexes fine-tune host metabolite function. This would fit well with the established view that sponge-associated microbes contribute heavily to sponge chemical ecology and that many bioactive metabolites isolated from sponges may actually originate from symbionts or symbiont-influenced pathways.

At the same time, this idea should be framed carefully. It is well supported that marine sponges host complex microbiomes and that *Streptomyces* and cyanobacterial associates occur in sponge systems. It is also well supported that boron-containing antibiotics exist in *Streptomyces*, *Nostoc*, myxobacteria, and related marine-associated systems. What is not yet directly proven is that a given sponge simultaneously contains identified *Streptomyces*, *Nostoc*, and myxobacterial symbionts that have each been shown in situ to produce specific boron-containing antibiotics and steroid–borate complexes. That stronger claim remains a hypothesis requiring metagenomics, metabolomics, imaging mass spectrometry, and in situ structural analysis. But as a conceptual model, it is highly compelling: the sponge holobiont is one of the few marine systems where boron-rich seawater, polyhydroxylated host steroids, and metabolically gifted microbial symbionts all converge in the same microenvironment [80,81,186,187,188,189,190,191,192,193].

## 7. Conclusions

The chemistry of boron–steroid interactions offers a compelling and largely untapped framework for understanding the structural diversity and biological behavior of marine polyhydroxylated steroids. Marine invertebrates—including sponges, echinoderms, corals, and their associated microorganisms—produce an extraordinary array of steroids characterized by dense hydroxylation patterns, vicinal diols, and 1,3-diol motifs embedded within rigid, preorganized steroidal scaffolds. From a coordination chemistry standpoint, these features make such metabolites intrinsically predisposed to bind boron, particularly in the form of tetrahedral borate, under the mildly alkaline and boron-rich conditions characteristic of marine environments.

Unlike classical steroid conjugates such as sulfates, glycosides, or fatty acid esters, boron–steroid complexes arise through non-enzymatic, reversible interactions that are highly sensitive to local physicochemical conditions. This reversibility allows marine polyhydroxy steroids to exist as dynamic ensembles of free and boron-bound species, rather than as single, static molecular entities. Such speciation provides a chemical mechanism for fine-tuning bioavailability, stability, and effective concentration in response to environmental cues such as pH fluctuations, changes in boron availability, or competition with other diol-containing ligands present in seawater, sediments, or biological matrices.

The examples discussed throughout this review—spanning sponges, starfish, soft corals, and marine-derived fungi—demonstrate that boron complexation is not an isolated curiosity but a recurring theme across diverse marine taxa and steroid classes. The number and nature of boron complex isomers are dictated not simply by hydroxyl count, but by precise hydroxyl positioning, stereochemistry, and conformational constraints within the steroid framework. Systems featuring well-defined *cis*-1,2-diols or preorganized diol arrays often yield a single dominant boron complex, whereas more flexible or highly oxygenated steroids can generate multiple boron-bound isomers. These observations underscore the central role of molecular topology in governing boron coordination behavior.

From a biological perspective, boron–steroid complexation provides a plausible explanation for longstanding inconsistencies in reported bioactivities of marine steroids. Variations in extraction solvents, pH, ionic composition, or purification conditions may shift the equilibrium between free and boron-bound forms, leading to substantial differences in observed potency, selectivity, or mechanism of action. In boron-enriched microenvironments—such as sponge mesohyls, mucus layers, coral tissues, or sediment-associated niches—boron complexes may function as stabilized storage forms, transport intermediates, or chemically buffered reservoirs of bioactive steroids. Upon release into boron-poor or acidic environments, dissociation of these complexes could regenerate the free, biologically active steroid.

The discovery of naturally occurring boron-containing metabolites, such as Territrem F borate, further reinforces the biological relevance of boron in marine secondary metabolism and highlights marine fungi as important contributors to this chemical space. Moreover, emerging evidence suggests that boron complexation can enhance or qualitatively alter biological activity, raising intriguing possibilities for the rational design of boron-based steroidal therapeutics inspired by marine natural products.

In a broader context, recognizing boron complexation as an integral component of marine steroid chemistry invites a shift in how these molecules are conceptualized, analyzed, and evaluated. Future studies should explicitly consider boron speciation during isolation, structural characterization, and bioassays, as well as explore the ecological and physiological roles of boron–steroid interactions in vivo. By bridging marine chemistry, coordination chemistry, and natural product biology, this perspective opens new avenues for understanding marine chemical ecology and for harnessing boron–steroid architectures in drug discovery. Ultimately, life with boron may represent not an exception, but a subtle and pervasive dimension of marine steroid biology that has only just begun to be appreciated.

Building on this perspective, two practical implications deserve particular emphasis. An important, often overlooked implication is that boron–steroid complexation is unlikely to be uniform across an organism or habitat, but instead concentrated in microenvironments where polyols accumulate and pH and ionic strength deviate from bulk seawater. In sponges and soft corals, such niches may include the mesohyl, canal systems, mucus layers, and symbiont-rich compartments, where local metabolite concentrations and constrained diffusion can shift equilibria toward borate-bound forms. In this view, “boronosteroids” are not necessarily rare covalent curiosities, but may represent a dynamic, context-dependent speciation layer superimposed on the classical steroid metabolome—an inorganic coordination regime capable of modulating solubility, membrane partitioning, and the effective concentration of the free bioactive steroid. This framework also offers a practical explanation for the frequently reported variability of marine steroid bioactivity across extraction conditions and assay media. Small changes in pH, buffering capacity, competing diols, or boron availability can shift the free/complexed ratio and thereby alter apparent potency without any change in covalent structure. A simple, testable prediction follows: under mildly alkaline conditions and physiologically relevant boron levels, selected polyhydroxysteroids may display boron-dependent shifts in chromatographic behavior, NMR signatures (including ^11^B), and membrane affinity, with reversibility upon acidification or competitive chelation. Systematic interrogation of this reversible coordination layer may therefore improve both mechanistic interpretation and reproducibility in marine natural product research.

## Figures and Tables

**Table 1:** DFT Energies of Stelletasterenol and Borate Complex.

Structure	Method	Energy (Hartree)	ΔE (kcal/mol)	HOMO (eV)	LUMO (eV)	HOMO–LUMO Gap (eV)	Dipole (D)
Stelletasterenol	M06-2X/def2-TZVP	−1523.4178	0	−6.21	−0.91	5.30	3.4
Stelletasterenol–borate complex	M06-2X/def2-TZVP	−1523.4629	−28.4	−6.35	−1.12	5.23	6.1

**Table 2:** Qualitative molecular-orbital comparison of stelletasterenol (**5**) and xestokerol A (**28**) and their proposed boron complexes.

Structure	Coordination Type	Expected HOMO Localization	Expected LUMO Localization	Expected Effect of Boron Coordination	Expected Relative Polarity
Stelletasterenol	Free molecule	Mainly on the hydroxyl-bearing region of the steroid nucleus, especially around O-2, O-3, and O-19	Distributed over the steroid framework and adjacent oxygenated regions	—	Moderate
Stelletasterenol–boron complex	Triol-borate complex involving O-2, O-3, and O-19	Reduced electron density on the coordinating oxygen atoms; HOMO shifted toward the remaining steroid framework	Slightly redistributed over the oxygenated ring system	Increased rigidity, stabilization of one major isomer, increased polarization	High
Xestokerol A	Free molecule	Mainly localized at the 3β-hydroxyl region and nearby carbon framework	Distributed over the sterol backbone, with possible contribution from the cyclopropyl-containing side chain	—	Low to moderate
Xestokerol A–boron complex	Monodentate or weak borate interaction at the 3β-hydroxyl site	Slight decrease in electron density at the hydroxyl oxygen; HOMO remains mainly steroid-centered	Minimal change, still dominated by the sterol skeleton	Small electronic perturbation; weaker effect than in polyhydroxylated sterols	Moderate

## Data Availability

No new data were created or analyzed in this study. Data sharing is not applicable to this article.
